# Human Health Effects of Chronic Microcystin Exposure: A Systematic Review and Meta-Analysis of Hepatic, Renal, and Metabolic Outcomes

**DOI:** 10.3390/toxins18090373

**Published:** 2026-08-30

**Authors:** Seong-Dae Moon, Dae-Sik Hwang, Jung-Suk Lee, Su-Ok Hwang, Hans W. Paerl, Baik-Ho Kim

**Affiliations:** 1 NEB Co., Seoul 08504, Republic of Korea; neb.sdmoon@gmail.com (S.-D.M.); neb.dshwang@gmail.com (D.-S.H.); neb.jslee@gmail.com (J.-S.L.); 2 Research Institute for Natural Sciences, Hanyang University, Seoul 04763, Republic of Korea; spring3974@naver.com; 3 Institute of Marine Sciences, Department of Earth, Marine and Environmental Sciences, University of North Carolina at Chapel Hill, Morehead City, NC 28557, USA; hans_paerl@unc.edu; 4 Department of Environmental Sciences and Engineering, University of North Carolina at Chapel Hill, Morehead City, NC 28557, USA; 5 Department of Environmental Science, Hanyang University, Seoul 04763, Republic of Korea

**Keywords:** microcystins, cyanotoxins, chronic exposure, human toxicology, systematic review, meta-analysis

## Abstract

Microcystins (MCs) are cyanobacterial hepatotoxins that contaminate bloom-affected freshwater, drinking-water sources, aquatic food webs, and recreational environments. Human evidence for chronic or repeated exposure remains fragmented across study designs and organ systems. We systematically reviewed epidemiological evidence for hepatic, renal, and metabolic outcomes and integrated supporting mechanistic toxicology. PubMed, Scopus, and Web of Science searches identified 9021 records published from 1990 to 2025; an update search was conducted in April 2026. Risk of bias was assessed using a domain-based framework adapted from the Newcastle–Ottawa Scale and OHAT. The review was not prospectively registered. The qualitative evidence architecture retained 192 studies: 34 human epidemiological studies and 158 supporting mechanistic or contextual studies. Only seven independent adjusted estimates met the one-study–one-main-estimate rule for the primary quantitative synthesis. The hepatic meta-analysis included two estimates (OR 2.13; 95% CI, 1.29–3.53), the renal finding was a descriptive single-study estimate (OR 4.81; 95% CI, 1.96–11.81), and the metabolic meta-analysis included four estimates (OR 1.73; 95% CI, 1.00–2.98) with substantial heterogeneity (I^2^ = 91%; prediction interval, 0.56–5.36). Mechanistic evidence supported biological plausibility but was not treated as causal human evidence. Human evidence was relatively more consistent for hepatic outcomes, although it remained limited to two independent studies with different endpoints; renal and metabolic findings remain sparse or heterogeneous.

## 1. Introduction

Cyanobacterial harmful algal blooms (CyanoHABs) threaten freshwater ecosystems, drinking-water safety, aquatic food webs, recreation, and public health [1]. Nutrient enrichment, altered hydrology, warming, and prolonged stratification can promote bloom formation and persistence [2,3,4,5,6,7,8,9]. Among cyanotoxins, microcystins (MCs) are of particular concern because they are produced by common bloom-forming taxa and occur in surface waters, drinking-water sources, aquatic food webs, and recreational environments [10,11,12,13,14].

Historically, MC risk assessment has focused mainly on acute hepatotoxicity. This focus is supported by evidence that the liver is the principal target organ of MC toxicity and by documented poisoning incidents, most notably the Caruaru dialysis tragedy, which demonstrated the fatal consequences of high-dose exposure [15,16]. Yet the Caruaru disaster—and the acute toxicity framework it reinforced—may have narrowed risk assessment to a scenario that does not reflect how most people encounter MCs. In communities near chronically blooming lakes, low-level exposure through tap water, locally caught fish, and recreational swimming can persist for months or years at a time [17,18,19]. Biomonitoring studies have further demonstrated the presence of MCs in serum and identified evidence of hepatocellular damage in chronically exposed populations, indicating that environmental exposure can result in measurable internal dose and biological effects [20].

An increasing number of epidemiological studies have investigated associations between chronic or repeated MC exposure and hepatic, renal, and metabolic outcomes. Reported endpoints include liver injury, hepatocellular carcinoma, chronic kidney disease, dyslipidemia, metabolic syndrome, and gestational diabetes [21,22,23,24,25,26,27]. Some associations appear stronger under co-exposure conditions involving cadmium, arsenic, aflatoxin, hepatitis virus infection, alcohol, or other hepatotoxic and nephrotoxic stressors. Such findings are particularly relevant because CyanoHAB-affected populations are rarely exposed to MCs in isolation. Consequently, interpretation of chronic MC-related health risks requires careful evaluation of exposure assessment, confounder adjustment, co-exposures, outcome definitions, and statistical dependence among multiple estimates reported from the same study population.

Mechanistic toxicology provides biological plausibility for evaluating MC-associated health effects beyond acute liver injury. MCs enter target cells via organic anion-transporting polypeptides and inhibit the protein phosphatases PP1 and PP2A, thereby triggering oxidative stress, cytoskeletal disruption, mitochondrial dysfunction, inflammatory signaling, and cellular injury [13,28,29,30]. Although the liver remains the central target organ, extrahepatic transporter expression and systemic inflammatory or metabolic responses provide plausible pathways linking MC exposure to renal and metabolic outcomes. Experimental and translational studies have also suggested links between MC exposure and renal tubular injury, metabolic disruption, and carcinogenic processes [31], supporting a broader, but cautious, multi-organ interpretation of the effects of chronic exposure [32,33,34].

Previous reviews have addressed MC occurrence, exposure routes, acute toxicity, mechanisms, monitoring, and risk management [10,12,13,14,18,35]. However, chronic human evidence remains fragmented across organ systems, study designs, exposure matrices, analytical methods, and outcome definitions, and few reviews have quantitatively integrated organ-specific epidemiological associations. A focused synthesis is therefore needed to distinguish the limited independent human evidence from the much larger mechanistic and contextual literature and to avoid treating these evidence streams as equivalent.

Exposure assessment also varies between environmental measurements and internal biomarkers and between screening assays and congener-specific analytical methods. These differences affect specificity, exposure-window interpretation, and comparability and are evaluated in detail in the Methods and Discussion.

To address these gaps, we conducted a toxin-centered systematic review and meta-analysis of human epidemiological evidence linking chronic or repeated MC exposure to hepatic, renal, and metabolic outcomes. The objectives were to: (i) identify and synthesize eligible human epidemiological studies; (ii) estimate outcome-specific associations while minimizing statistical dependence through a conservative one-study–one-main-estimate rule; (iii) evaluate mechanistic coherence across MC uptake, PP1/PP2A inhibition, oxidative and mitochondrial injury, inflammatory signaling, and organ-specific toxicity; and (iv) identify limitations in exposure characterization, biomonitoring, congener confirmation, and mixture-aware interpretation. Environmental and public-health evidence was used only to contextualize exposure and risk translation and was not treated as primary causal evidence. The problem formulation is presented in the Materials and Methods section.

## 2. Results

### 2.1. Study Selection and Evidence Architecture

The searches identified 9021 records. After 1158 duplicates were removed, 7863 records underwent title–abstract screening; 6405 were excluded and 1458 full texts were assessed. The qualitative evidence architecture retained 192 studies: 34 human epidemiological studies and 158 supporting studies (86 mechanistic/translational, 54 environmental/exposure-pathway, 6 economic/societal, 6 governance/monitoring, and 6 vulnerability/implementation studies). Only the 34 human studies addressed the primary human-health review question; the remaining 158 studies were used solely to evaluate biological plausibility or contextualize exposure and risk translation. The category-level distribution is shown in Supplementary Table S3.

**Figure 1:**
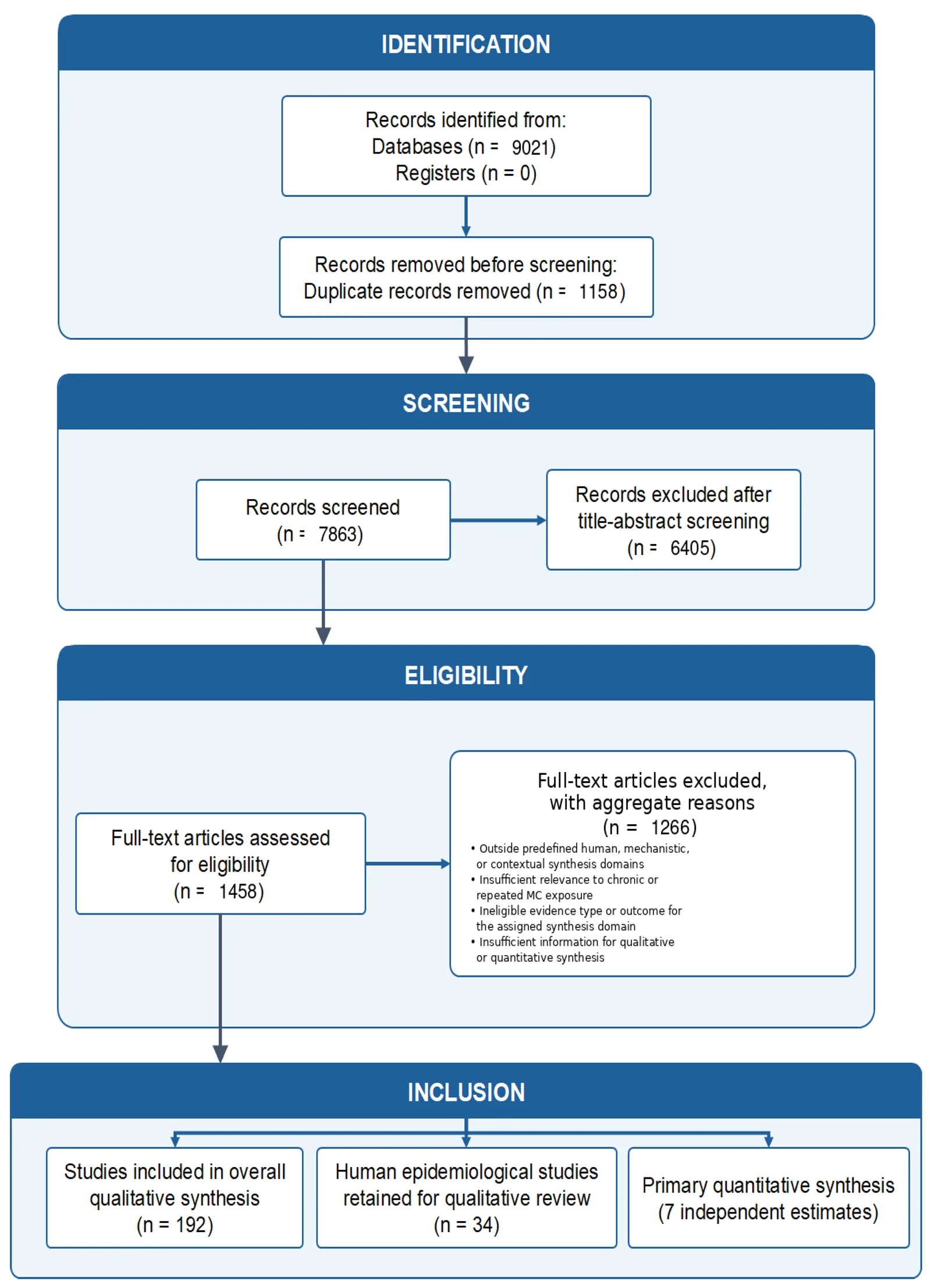
PRISMA 2020 flow diagram for study identification, screening, eligibility assessment, and inclusion. Searches identified 9021 records; 7863 unique records were screened and 1458 full-text articles were assessed. Overall, 192 studies were retained in the qualitative evidence architecture, comprising 34 human epidemiological studies and 158 supporting mechanistic or contextual studies. Seven independent adjusted human epidemiological estimates contributed to the primary quantitative synthesis. The flow diagram reports aggregate full-text exclusion reasons because a study-level exclusion log was not retained.

Among the 34 human epidemiological studies retained for qualitative review, seven independent adjusted estimates met the criteria for the primary outcome-specific quantitative synthesis under the conservative one-study–one-main-estimate rule. The separation among 192 retained studies, 34 human studies, and seven primary estimates is shown explicitly in Figure 1 and Supplementary Table S3. An itemized list of full-text exclusions could not be reconstructed from the retained review files; aggregate reasons are reported, and this reporting limitation is acknowledged in Section 3.5 and the Supplementary Materials.

The final evidence architecture was organized into three levels: primary human epidemiological evidence, supporting mechanistic evidence, and contextual environmental, governance, and vulnerability-related evidence. Only eligible adjusted human epidemiological estimates were included in the primary quantitative meta-analysis.

### 2.2. Characteristics of Human Epidemiological Studies

The seven independent primary estimates selected for the main quantitative synthesis varied in study design, geographic setting, exposure assessment, outcome definition, and covariate adjustment. Most primary human evidence came from regions with established environmental monitoring or biomonitoring capacity, particularly China, where serum, plasma, drinking water, dietary, or environmental MC measurements have been linked to individual-level health outcomes. Study designs included cross-sectional, case–control, nested case–control, and population-based observational designs. Longitudinal cohort evidence remained limited.

Exposure assessment differed across studies. Several investigations used biomonitoring matrices, including serum or plasma MC measurements, whereas others relied on drinking-water, dietary, or inferred environmental exposure categories. Analytical methods included ELISA-based assays and more specific analytical chemistry methods, including mass spectrometry, where available. These differences were considered important sources of heterogeneity and exposure-specific uncertainty.

Outcome groups were classified as hepatic, renal, and metabolic. Hepatic outcomes included liver injury markers and hepatocellular carcinoma. Renal outcomes included chronic kidney disease and renal injury indicators. Metabolic outcomes included dyslipidemia, metabolic syndrome, lipid-profile abnormalities, and gestational diabetes. Adjustment for major confounding factors varied across outcome groups, particularly with respect to hepatitis virus infection, aflatoxin exposure, alcohol use, smoking, body mass index, hypertension, diabetes, socioeconomic factors, pregnancy-related variables, and co-exposure to metals or other environmental contaminants.

For the primary quantitative synthesis, the selected main estimate represented the most fully adjusted population-level estimate for each parent study and outcome group. Subgroup, dose–response, interaction, co-exposure, mediation, sensitivity, review-derived, and contextual estimates were extracted separately and were not treated as independent primary observations. Study-level characteristics, selected adjusted estimates, risk-of-bias assessments, and synthesis roles of the records included in the primary quantitative synthesis are summarized in Table 1. Detailed extracted estimates and estimate-selection procedures are provided in Supplementary Table S5.

### 2.3. Epidemiological Evidence by Outcome Group

Human epidemiological evidence was grouped as hepatic, renal, or metabolic because these outcomes differ in clinical definition, exposure window, latency, and confounding structure. The primary evidence comprised two hepatic estimates, one descriptive renal estimate, and four metabolic estimates; detailed dose–response, subgroup, co-exposure, mediation, sensitivity, and excluded estimates are provided in Supplementary Table S5.

Hepatic studies evaluated liver injury and hepatocellular carcinoma. Renal evidence comprised one main CKD estimate, with metal co-exposure contrasts retained only as secondary analyses. Metabolic studies evaluated dyslipidemia, metabolic syndrome, and gestational diabetes and produced inconsistent study-level results.

Joint-exposure results involving arsenic, cadmium, aflatoxin, or alcohol were treated as potential effect-modification signals but could also reflect residual confounding; they were not interpreted as proof of mixture effects or pooled as independent observations.

Because subgroup and interaction estimates were statistically dependent on their parent studies, they were retained only as secondary evidence.

### 2.4. Quantitative Synthesis, Heterogeneity, and Sensitivity Analyses

The quantitative synthesis distinguished between independent primary estimates and the broader set of extracted effect-size estimates. Seven independent adjusted estimates were eligible for the main outcome-specific quantitative synthesis. The expanded extracted-estimate set included additional estimates from dose–response categories, subgroup analyses, interaction terms, co-exposure analyses, mediation analyses, sensitivity analyses, and alternative outcome definitions. To reduce statistical dependence and avoid over-weighting parent studies that reported multiple related estimates, the primary synthesis followed the predefined one-study–one-main-estimate rule.

**Figure 2:**
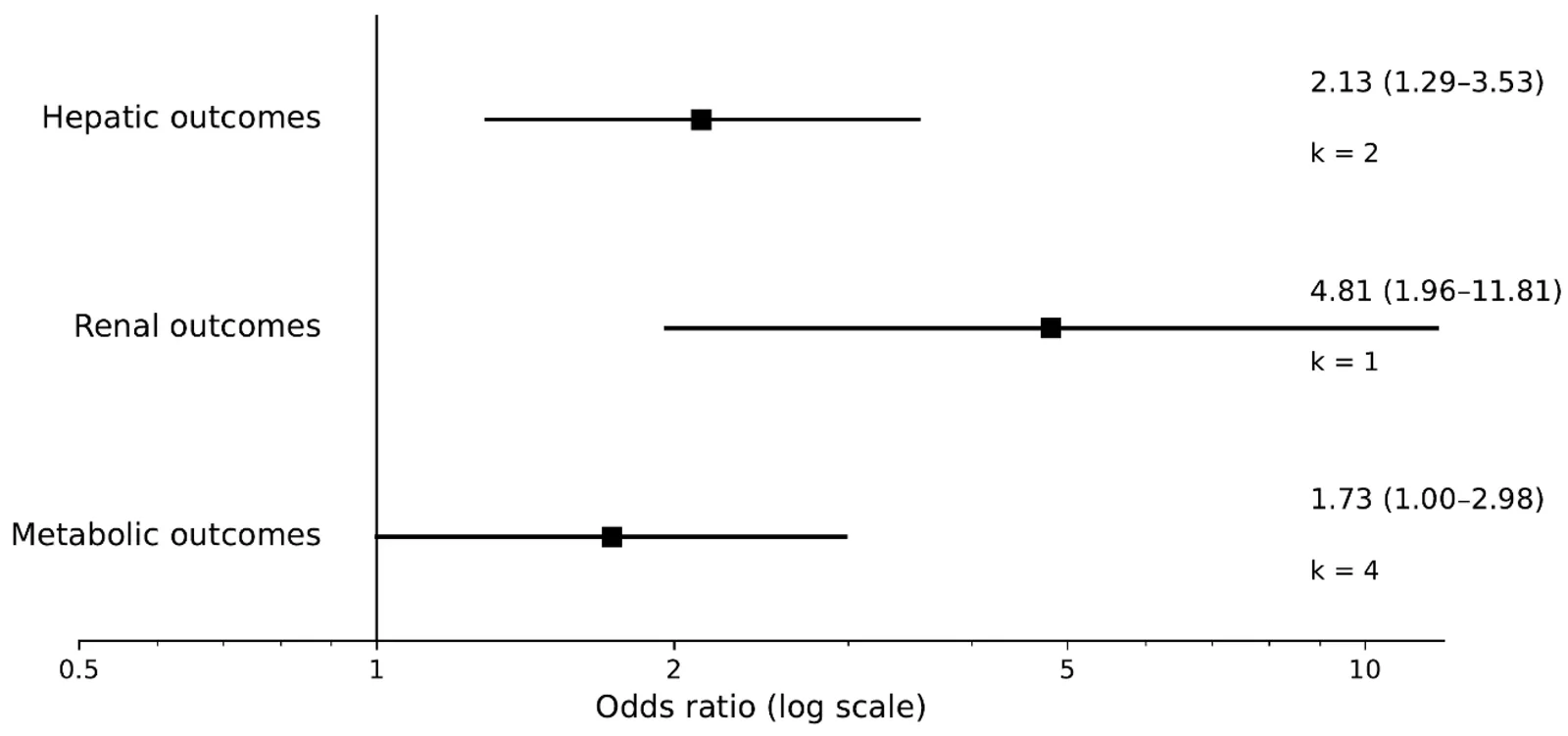
Outcome-specific display of chronic microcystin exposure and human hepatic, renal, and metabolic outcomes. Hepatic and metabolic markers represent pooled estimates; the renal marker is explicitly a descriptive estimate from one study and is not a meta-analytic summary. Horizontal bars indicate 95% confidence intervals. Hepatic outcomes yielded OR 2.13 (95% CI, 1.29–3.53; k = 2), the single renal study yielded OR 4.81 (95% CI, 1.96–11.81; k = 1), and metabolic outcomes yielded OR 1.73 (95% CI, 1.00–2.98; k = 4; I^2^ = 91%; prediction interval, 0.56–5.36).

Outcome-specific primary models are summarized in Figure 2 and Table 2. Hepatic outcomes yielded OR 2.13 (95% CI, 1.29–3.53; k = 2), renal outcomes were represented by a descriptive single-study estimate of OR 4.81 (95% CI, 1.96–11.81; k = 1), and metabolic outcomes yielded OR 1.73 (95% CI, 1.00–2.98; k = 4). Because only one independent renal estimate was available, no renal pooled model or heterogeneity statistic was calculated. The exploratory cross-outcome comparison and the expanded extracted-estimate analysis are reported separately in Table 2 and Supplementary Figure S2 and were not used as primary inferential results.

The expanded 42-estimate comparison was retained only as an exploratory sensitivity analysis in Supplementary Figure S2. Because multiple estimates originated from the same parent studies and were statistically dependent, it is not presented as a pooled result in the main text or Table 2 and should not be used for inference.

Heterogeneity differed across outcome groups. Heterogeneity was low for hepatic outcomes, could not be assessed for the single-estimate renal summary, and was substantial for metabolic outcomes. Metabolic heterogeneity appeared to reflect differences in endpoint definition, exposure thresholds, population characteristics, analytical methods, and covariate adjustment. Sensitivity analyses evaluated the influence of high-risk-of-bias estimates, biomonitoring-based exposure assessment, analytically validated exposure measurements, non-OR effect metrics, subgroup or interaction estimates, and co-exposure or mediation estimates. Full sensitivity, influence, small-study-effect, and diagnostic analyses are provided in Supplementary Figure S2.

Overall, the primary outcome-specific analyses provided the strongest interpretive support for hepatic outcomes. The renal result was a descriptive single-study signal, and the metabolic summary was borderline and substantially heterogeneous. Exploratory cross-outcome and expanded extracted-estimate comparisons were retained only for sensitivity and transparency and were not interpreted as primary pooled health-effect estimates.

### 2.5. Mechanistic Toxicology and Exposure Context

**Figure 3:**
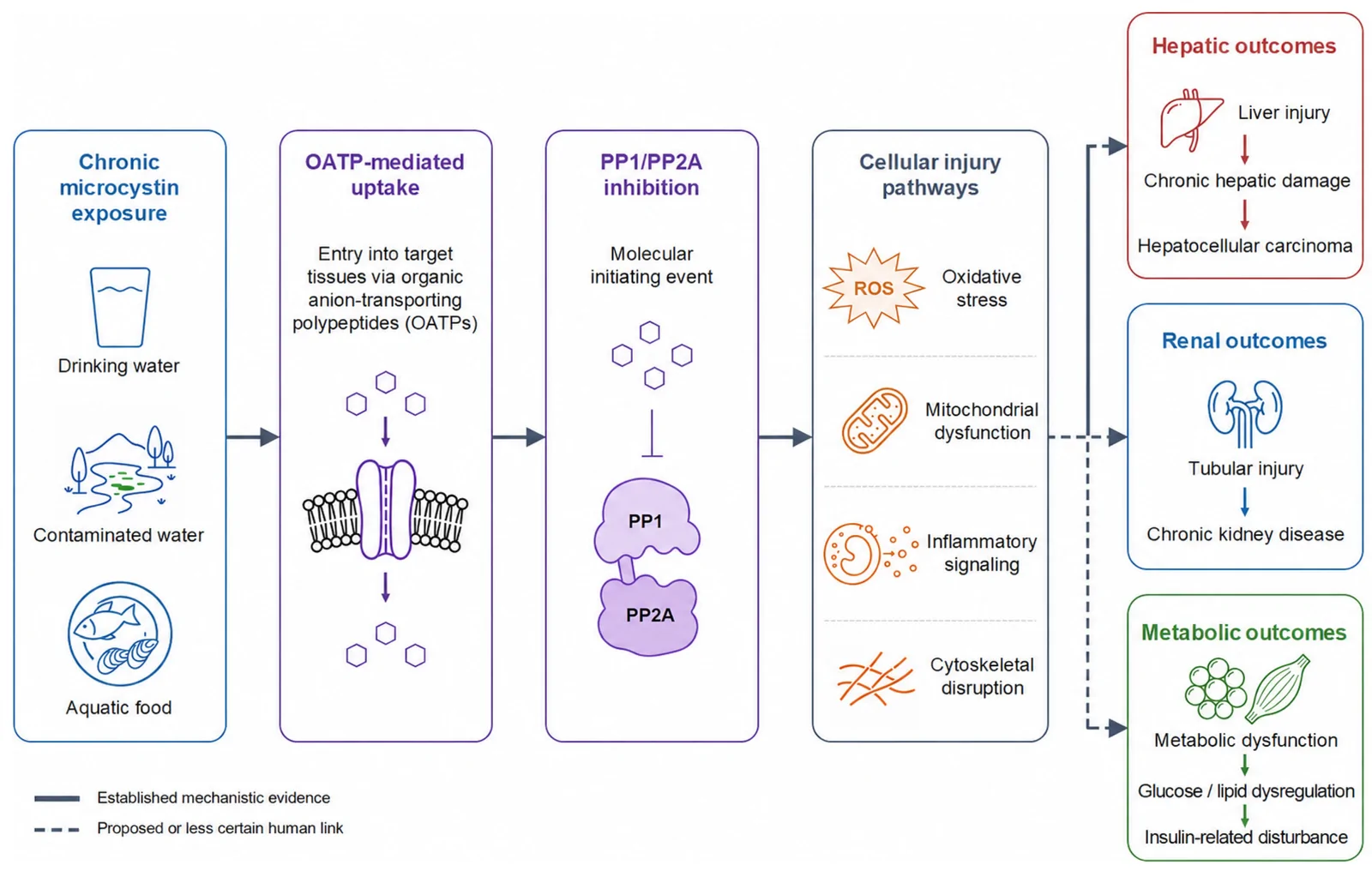
Mechanistic coherence linking microcystin uptake to organ-level outcomes. Solid arrows denote established mechanistic events and the comparatively better-supported hepatic pathway. Dashed arrows denote proposed or less certain human links to renal and metabolic outcomes. The schematic supports interpretation of biological plausibility and does not establish causality.

Mechanistic studies were synthesized to evaluate biological coherence with the epidemiological outcome groups. Figure 3 summarizes the sequence from environmental or biomonitoring-based exposure to OATP-mediated uptake, PP1/PP2A inhibition, oxidative and mitochondrial injury, inflammatory signaling, and hepatic, renal, or metabolic outcomes. Supporting evidence on exposure routes, analytical methods, and monitoring context is summarized in Table 3 and was used only to interpret exposure plausibility and toxicological uncertainty.

## 3. Discussion

### 3.1. Principal Findings

This review found relatively more consistent human evidence for hepatic outcomes than for renal or metabolic outcomes, but the absolute evidence base remained small. The hepatic meta-analysis contained only two independent studies with different endpoints; the renal result was a descriptive single-study signal; and the metabolic meta-analysis was based on four studies with high heterogeneity and a wide prediction interval. Accordingly, the pooled values should be interpreted as limited outcome-specific summaries rather than precise estimates of generalized multi-organ risk.

The seven primary estimates represent a conservative subset of the 34 human studies and should not be conflated with the 192-study qualitative architecture. Additional dependent estimates were retained for transparency in the Supplementary Materials but were not allowed to increase the apparent precision of the primary analyses.

Hepatic associations were biologically coherent but remain vulnerable to residual confounding and exposure misclassification. The renal and metabolic findings warrant further study but currently provide insufficient evidence for strong causal or cross-organ conclusions.

Taken together, the available evidence suggests that chronic MC exposure may affect health outcomes beyond acute hepatotoxicity; however, current epidemiological evidence is insufficient to quantify multi-organ risks with high certainty across diverse exposure settings.

### 3.2. Outcome-Specific Interpretation: Hepatic, Renal, and Metabolic Evidence

Relative to the other outcome groups, hepatic outcomes were better supported. However, this judgment is comparative rather than an indication of strong absolute certainty: only Li et al. [21] and Zheng et al. [22] contributed independent primary hepatic estimates, and they evaluated different endpoints (liver injury and hepatocellular carcinoma). Residual confounding by hepatitis infection, aflatoxin, alcohol, metabolic liver disease, and socioeconomic conditions remains possible.

Renal evidence was limited to one independent primary CKD estimate. Stronger associations reported under cadmium or arsenic co-exposure [23,24] may indicate effect modification, but they may also reflect residual confounding or correlated exposures. These joint contrasts therefore do not demonstrate a mixture effect and were retained only as secondary evidence.

Metabolic evidence remained inconsistent. Four studies yielded a borderline pooled estimate with high heterogeneity (I^2^ = 91%) and a prediction interval spanning the null (0.56–5.36). Individual studies differed in outcomes, exposure thresholds, populations, assays, and covariate adjustment; therefore, the study-level results and sources of heterogeneity are more informative than the pooled value alone. The relationship between metabolic dysfunction and liver injury also complicates outcome interpretation [36].

### 3.3. Exposure Assessment, Heterogeneity, and Co-Exposure Complexity

Differences between environmental proxies and serum or urine biomarkers, and between ELISA screening and LC–MS/MS confirmation, likely contributed to exposure misclassification and heterogeneity. These approaches represent different exposure windows and analytical specificity; future studies should combine repeated biomonitoring with congener-specific confirmation where feasible.

Co-exposure also complicates interpretation. Stronger associations in populations exposed to arsenic, cadmium, aflatoxin, alcohol, or other stressors may reflect genuine effect modification, residual confounding, or both. Joint-exposure contrasts were therefore kept separate from MC main effects and were not treated as proof of mixture toxicity. This mixture-aware interpretation is consistent with an exposome framework [37].

These differences do not dismiss the associations, but they limit how broadly the results can be interpreted. The most informative studies were those with repeated or individual-level exposure assessment, validated MC measurements, clinically or biomarker-defined outcomes, clear co-exposure assessment, and outcome-specific confounder adjustment. Future epidemiological studies would benefit from harmonized biomonitoring strategies and standardized analytical approaches to improve comparability across populations and regions.

### 3.4. Mechanistic Interpretation

Mechanistic evidence provides important biological coherence for the epidemiological associations observed in this review. MCs can enter target tissues through organic anion-transporting polypeptides, particularly OATP-family transporters, and inhibit PP1/PP2A, initiating oxidative stress, cytoskeletal disruption, mitochondrial dysfunction, inflammatory signaling, and cellular injury [13,28,29,30]. As illustrated in Figure 3, these pathways provide a plausible bridge between environmental or biomonitoring-based MC exposure and hepatic, renal, and metabolic outcomes.

The hepatic pathway is the most established mechanistic axis because the liver is the primary target organ of MC toxicity. Efficient hepatocellular uptake and PP1/PP2A inhibition are consistent with cytoskeletal collapse, oxidative damage, inflammatory signaling, and tumor-promoting processes [28,29,30]. Renal and metabolic mechanisms are also biologically plausible, although the supporting evidence remains less established. Extrahepatic transporter expression, renal tubular uptake, mitochondrial injury, inflammatory responses, and disruption of lipid or glucose regulation may contribute to renal and metabolic outcomes, but translation from animal or cellular models to chronic, low-dose human exposure remains uncertain [32,33,34,38].

Mechanistic coherence should therefore not be confused with causal proof in humans. Experimental toxicology often relies on specific congeners, controlled exposure conditions, higher exposure levels, or short experimental windows that do not fully reproduce chronic, multi-route human exposure [13,30]. Mechanistic evidence was therefore not pooled with human epidemiological evidence and was not used as a substitute for human outcome data. Its primary role was to support biological plausibility and strengthen the interpretation of the epidemiological findings rather than establish causality.

### 3.5. Limitations and Priorities for Chronic Microcystin Toxicology

This review has several limitations. First, the human epidemiological evidence base remains geographically concentrated, with much of the quantitative evidence derived from regions where environmental monitoring, biomonitoring, and linked health data are relatively well developed. This should be interpreted as a limitation of surveillance rather than as evidence that chronic MC-related health risks are geographically restricted. Global reviews of inland HABs emphasize that bloom occurrence, drivers, monitoring capacity, and mitigation needs are unevenly distributed across regions [11,39].

Second, most of the included studies were observational and heterogeneous in their exposure assessment, outcome definition, and confounder adjustment. Longitudinal cohort evidence remains limited, restricting inference about latency, temporality, and causal direction. Third, exposure misclassification remains a major concern because serum or urine biomarkers, drinking-water measurements, food-web exposure, recreational contact, aerosol exposure, and environmental proxies capture different exposure windows [17,18,19]. Fourth, residual confounding and co-exposure remain important, especially for hepatic outcomes affected by hepatitis virus infection, aflatoxin, alcohol, and metabolic liver disease; renal outcomes affected by hypertension, diabetes, cadmium, and arsenic; and metabolic outcomes affected by body mass index, diet, pregnancy-related factors, and socioeconomic conditions.

Fifth, the exact original database export logs and an itemized list of the 1266 full-text exclusions were not retained. Consequently, Supplementary Table S2 reports reconstructed database-adapted syntax, while Figure 1 and the Supplementary Materials provide only aggregate exclusion reasons. This limits full auditability of the selection process and is explicitly acknowledged rather than reconstructed retrospectively.

These limitations define priorities for chronic MC toxicology and exposure science. Future studies should prioritize prospective or repeated-measures designs, repeated human biomonitoring, LC-MS/MS confirmation and congener-specific quantification where feasible, standardized hepatic, renal, and metabolic endpoints, and explicit assessment of co-exposures. Studies should link environmental occurrence, external exposure, internal dose, molecular initiating events, and clinically relevant outcomes. Such integration is needed to determine whether associations observed in current epidemiological studies reflect causal toxin effects, mixture-related susceptibility, or residual exposure and outcome misclassification.

## 4. Conclusions

Current human evidence suggests possible associations between chronic or repeated MC exposure and hepatic, renal, and metabolic outcomes, but it remains too small and heterogeneous to support a generalized multi-organ risk estimate. Hepatic findings were relatively more consistent but were based on only two independent studies with different endpoints. Renal evidence comprised one descriptive estimate, and metabolic findings were heterogeneous with a prediction interval spanning the null. Prospective cohorts, repeated and analytically validated biomonitoring, congener-specific confirmation, harmonized outcome definitions, and explicit co-exposure assessment are needed before stronger causal conclusions can be drawn.

## 5. Materials and Methods

### 5.1. Review Question, Protocol Transparency, and PECO Framework

This systematic review and meta-analysis was prepared and reported in accordance with the Preferred Reporting Items for Systematic Reviews and Meta-Analyses 2020 statement [40]. Because the review addresses environmental exposure and human health outcomes, the problem formulation and evidence-synthesis structure were also informed by good-practice principles for environmental health systematic reviews, including the Navigation Guide, the OHAT approach, and the COSTER recommendations for systematic reviews in toxicology and environmental health research [41,42,43].

The review question was structured using a Population–Exposure–Comparator–Outcome (PECO) framework [44]: in human populations, is chronic or repeated exposure to microcystins (MCs) associated with hepatic, renal, or metabolic health outcomes compared with lower or no exposure? The population of interest comprised individuals with measured or inferred environmental exposure to MCs. The exposure was chronic or repeated MC exposure through drinking water, aquatic food webs, recreational water, aerosols, or biomonitoring matrices such as serum or urine. The comparator was a lower-exposure, unexposed, or reference group as defined in each eligible study. Outcomes included hepatic, renal, and metabolic endpoints, including clinically diagnosed disease, ICD-coded outcomes, and validated biomarkers of organ injury or metabolic dysfunction.

**Figure 4:**
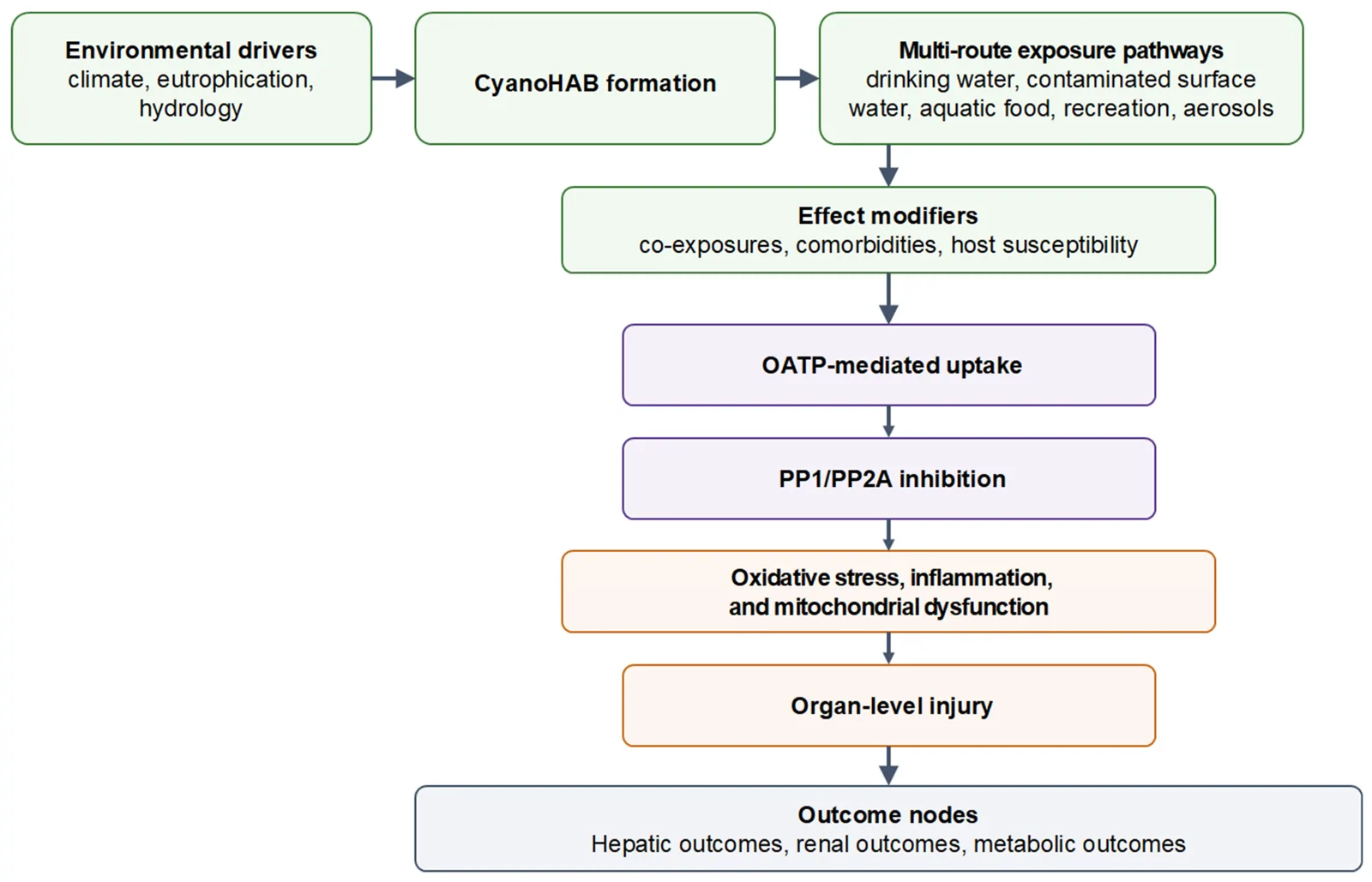
Toxin-centered problem-formulation framework for chronic microcystin exposure and hepatic, renal, and metabolic human health outcomes. This framework links environmental drivers and CyanoHAB formation to multi-route microcystin exposure, OATP-mediated uptake, PP1/PP2A inhibition, oxidative and mitochondrial injury, inflammatory signaling, organ-level toxicity, and hepatic, renal, and metabolic outcomes. Host susceptibility, co-exposures, and comorbidities may modify progression across these steps. The framework organizes toxicological and epidemiological evidence and does not itself establish causality.

Figure 4 presents the conceptual problem-formulation map to align the PECO framework with exposure pathways, mechanistic plausibility, and the outcome groups evaluated in this review, conceptually grounded in the Adverse Outcome Pathway (AOP) framework [45,46]. The framework was used solely to organize the evidence and clarify hypothesized pathways; it was not treated as independent causal evidence.

Registration: This systematic review was not prospectively registered in PROSPERO, the Open Science Framework (OSF), INPLASY, or another public registry; therefore, no registration number is available. A prospective public protocol was not registered before the initial review. To improve transparency, the Supplementary Materials document the reconstructed database-adapted search strategy, PECO eligibility rules, standardized extraction fields, risk-of-bias judgments, and effect-size selection decisions. They also distinguish the primary human epidemiological synthesis from supporting mechanistic evidence and contextual public-health evidence.

### 5.2. Search Strategy and Study Identification

We searched PubMed, Scopus, and Web of Science for studies published between 1 January 1990 and 1 November 2025. A final update search was conducted in April 2026 using the same PECO-based strategy. Search reporting was guided by PRISMA-S and checked against PRESS principles [47,48]. A retrospectively reconstructed, database-adapted search strategy, field syntax, documented search timing, and evidence-domain mapped counts are provided in Supplementary Table S2. Because verbatim database export histories were not retained, the supplementary search report distinguishes reconstructed query syntax from the raw 9021-record PRISMA identification total.

The search strategy was developed around four core concepts: MCs and CyanoHABs; chronic or repeated environmental exposure; human hepatic, renal, or metabolic outcomes; and epidemiological effect measures. The search included both controlled vocabulary and free-text terms related to microcystin, cyanotoxin, cyanobacterial bloom, drinking water, food-web exposure, aerosol exposure, biomonitoring, liver injury, hepatocellular carcinoma, chronic kidney disease, dyslipidemia, metabolic syndrome, diabetes, odds ratio, risk ratio, hazard ratio, cohort, case–control, cross-sectional, and epidemiology. The search concepts, executed search strings, search dates, and evidence-domain mapping are provided in Supplementary Table S2.

To reduce the risk of missing key evidence, the strategy was checked against sentinel studies on human MC biomonitoring, liver injury, hepatocellular carcinoma, chronic kidney disease, dyslipidemia, metabolic syndrome, and gestational diabetes, including Chen et al. [20], Li et al. [21], Zheng et al. [22], Feng et al. [23], Gao et al. [24], Feng et al. [25], Lin et al. [26], and Feng et al. [27]. Reference lists of eligible studies and relevant reviews were also screened.

Regulatory and guidance documents from the WHO, U.S. EPA, OECD, and related agencies were used to provide contextual interpretation of exposure assessment, guideline values, monitoring practice, and risk-management implications. These documents were not included in the quantitative meta-analysis unless they reported primary human epidemiological effect estimates eligible under the PECO framework. Retrieved records were imported into a reference management database, deduplicated using DOI, title, author, and publication year matching, and screened using Rayyan [49]. The overall study-selection process is summarized in the PRISMA flow diagram.

### 5.3. Eligibility Criteria, Screening, and Data Extraction

Studies were eligible for the primary epidemiological synthesis if they met all of the following criteria: human populations; measured or inferred chronic or repeated MC exposure; comparison between higher and lower exposure groups; hepatic, renal, or metabolic outcome; observational epidemiological design; and adjusted relative-effect estimate with 95% confidence interval or sufficient adjusted information to derive one. Eligible designs included cohort, case–control, cross-sectional, panel, and time-series studies. Studies reporting only unadjusted estimates were retained for narrative or contextual interpretation when relevant, but were excluded from primary quantitative synthesis.

Studies were excluded from the primary quantitative synthesis if they were animal experiments, in vitro studies, mechanistic studies without human health outcomes, ecological country-level analyses, review articles, editorials, commentaries, acute poisoning case reports without chronic or repeated exposure information, or economic and governance studies without individual- or population-level human health effect estimates. Mechanistic toxicology studies were retained only as supporting evidence for biological plausibility. Environmental, governance, economic, and equity-related studies were retained as contextual evidence when they informed exposure interpretation, surveillance needs, or public-health evidence gaps.

Title–abstract screening and full-text review were conducted independently by two reviewers. Before formal screening, reviewers pilot-tested the eligibility criteria on a subset of records to harmonize interpretation of exposure, comparator, outcome, and study-design boundaries. Disagreements were resolved by discussion, and unresolved cases were adjudicated by a third reviewer. Reasons for full-text exclusion were recorded and summarized in the PRISMA flow diagram.

Data extraction was performed using a standardized codebook developed before final extraction. Extracted variables included study identifier, country and region, study design, population characteristics, exposure matrix, analytical method, exposure definition, comparator definition, outcome definition, outcome ascertainment, effect metric, adjusted covariates, co-exposures, subgroup or interaction status, mediation or sensitivity status, and risk-of-bias domains. Data were extracted by one reviewer and independently verified against the source article by a second reviewer.

For the primary quantitative synthesis, we prioritized the most fully adjusted main population-level estimate for each parent study and outcome group. When multiple estimates were available from the same study, we selected the estimate corresponding to the primary population-level analysis and the most complete confounder adjustment. Subgroup, dose–response, interaction, co-exposure, mediation, sensitivity, review-derived, and contextual estimates were extracted separately and retained for secondary or narrative interpretation; however, they were not considered independent observations in the primary quantitative synthesis.

### 5.4. Risk-of-Bias and Certainty Assessment

Risk of bias in human epidemiological studies included in the primary synthesis was assessed using a domain-based approach adapted from the Newcastle-Ottawa Scale [50] and the OHAT risk-of-bias tool and the Navigation Guide framework for environmental health evidence synthesis [41,42,43,51]. Summary quality scores were not used because total scores can obscure domain-specific threats to internal validity, particularly exposure misclassification and residual confounding.

Each eligible study–outcome estimate was assessed across participant selection, exposure assessment, outcome assessment, confounding, temporality, missing data, selective reporting, and statistical analysis. Two reviewers independently assessed each domain, with disagreements resolved by discussion or third-reviewer adjudication. Exposure assessment, confounding control, outcome assessment, and temporality were treated as key domains.

Exposure-assessment judgments considered exposure matrix, analytical method, validation status, temporal coverage, and risk of exposure misclassification. Studies using validated biomonitoring or analytically confirmed exposure assessments were judged more favorably than studies relying solely on ecological proxies, limited water measurements, or insufficiently described ELISA-based classifications. Outcome-assessment judgments considered whether hepatic, renal, or metabolic endpoints were clinically diagnosed, ICD-coded, registry-based, biomarker-based, or self-reported.

Confounding judgments were outcome-specific. For hepatic outcomes, critical covariates included age, sex, hepatitis virus infection, alcohol use, aflatoxin exposure, smoking, socioeconomic factors, and major liver disease risk factors. For renal outcomes, critical covariates included age, sex, hypertension, diabetes, baseline renal function when available, socioeconomic factors, and nephrotoxic co-exposures such as cadmium or arsenic. For metabolic outcomes, critical covariates included age, sex, body mass index, smoking, diet or lifestyle factors when available, socioeconomic factors, pregnancy-related variables where relevant, and metal or contaminant co-exposures.

Overall risk of bias was derived from domain-level judgments rather than numerical scoring. Ratings were summarized at the study–outcome estimate level and incorporated into interpretation through sensitivity analyses and, where sufficient data were available, subgroup analyses by exposure-assessment method. Risk-of-bias ratings were not used as statistical weights.

The certainty of evidence was assessed separately for hepatic, renal, and metabolic outcome groups using a GRADE-informed framework adapted for environmental and occupational health evidence synthesis [52,53,54]. Because the quantitative human evidence was observational, the initial certainty rating was low. Certainty could be downgraded for serious risk of bias, inconsistency, indirectness, imprecision, or suspected publication bias. It could be upgraded for consistent exposure–response patterns, large magnitude of association, improved exposure specificity, or coherent mechanistic support. Mechanistic toxicology evidence was not pooled with human epidemiological evidence and was not used as a substitute for human outcome data.

### 5.5. Data Synthesis and Meta-Analysis

The primary quantitative synthesis was limited to human epidemiological studies that reported adjusted effect estimates for chronic or repeated MC exposure and hepatic, renal, or metabolic outcomes. Mechanistic, animal, in vitro, ecological, economic, governance, and review studies were summarized separately as supporting or contextual evidence and excluded from the quantitative meta-analysis.

The primary quantitative synthesis was based on seven independent adjusted estimates selected using a conservative one-study–one-main-estimate rule to reduce statistical dependence. When multiple estimates were available from the same parent study or outcome group, we selected the most fully adjusted main population-level estimate comparing the highest or clearly elevated MC exposure category with the lowest or reference category. Subgroup, interaction, co-exposure, mediation, and sensitivity estimates were retained only for secondary interpretation.

The broader extracted-estimate dataset was retained for secondary interpretation, including dose–response description, subgroup patterns, evidence of co-exposure and interactions, mediation findings, robustness assessment, and contextual evidence mapping. These additional estimates were not treated as independent primary observations. Co-exposure estimates, including MC–cadmium, MC–arsenic, MC–alcohol, and MC–hepatitis-related interaction estimates, were interpreted as evidence of effect modification or mixture-related vulnerability rather than as independent main effects of MC exposure.

Effect estimates were harmonized on the natural-log relative-effect scale. Odds ratios were used as the primary effect metric. Standard errors were calculated from 95% confidence intervals using SE = [ln(upper CI) − ln(lower CI)]/3.92. When necessary, estimates were reoriented so that values greater than 1.0 consistently indicated higher risk among populations with higher MC exposure.

Random-effects meta-analyses were fitted using restricted maximum likelihood estimation in R version 4.5.1 (R Foundation for Statistical Computing, Vienna, Austria) with the metafor package [55]. Separate primary models were estimated for hepatic, renal, and metabolic outcome groups because these endpoints differ biologically, clinically, and methodologically. A cross-outcome pooled estimate based only on the selected primary estimates was calculated as a sensitivity comparison, not as the main inferential model. Pooled estimates were reported as odds ratios with 95% confidence intervals and, where appropriate, 95% prediction intervals. Between-study heterogeneity was quantified using τ^2^, I^2^, and the Q statistic [56], with interpretation focused on the magnitude and sources of heterogeneity rather than statistical significance alone.

The expanded extracted-estimate comparison was retained only as a secondary exploratory analysis. It was not used for primary inference because several parent studies contributed multiple statistically dependent estimates. Where data were sufficient, dependence among extracted estimates was examined using parent-study clustering or sensitivity analyses; otherwise, the expanded comparison was interpreted only as a directional summary of the broader extracted evidence base.

Potential sources of heterogeneity were explored according to outcome group, exposure matrix, analytical method, study design, geographic region, exposure-assessment specificity, and risk-of-bias category. Meta-regression was performed only when there were sufficient estimates to support stable modeling, with approximately 10 estimates per covariate as a minimum threshold; otherwise, heterogeneity was interpreted narratively.

Publication bias and small-study effects were evaluated using contour-enhanced funnel plots and Egger’s regression [57] when at least 10 effect estimates were available for a given model. Influence diagnostics, including leave-one-out analysis, studentized residuals, Cook’s distance, and Baujat plots [58] where applicable, were used to identify influential estimates and major contributors to heterogeneity [58,59]. Outliers were not removed solely on statistical grounds; their influence was evaluated through sensitivity analyses and interpreted in relation to study design, exposure assessment, outcome definition, co-exposure status, and risk of bias.

Sensitivity analyses evaluated robustness of the pooled estimates by excluding high-risk-of-bias estimates, restricting to biomonitoring-based exposure assessment, restricting to analytically validated exposure measurements where available, excluding subgroup, interaction, mediation, or co-exposure estimates, excluding non-OR effect metrics, applying the one-study–one-main-estimate rule, and comparing primary outcome-specific models with the expanded exploratory comparison.

Mechanistic toxicology evidence was synthesized narratively to assess biological plausibility and coherence with the epidemiological findings. This synthesis focused on OATP-mediated uptake, PP1/PP2A inhibition, oxidative stress, mitochondrial dysfunction, inflammatory signaling, and organ-specific pathways relevant to hepatic, renal, and metabolic outcomes. Environmental, governance, and vulnerability-related evidence was used only to contextualize exposure prevention and surveillance needs and was not incorporated into the quantitative meta-analysis or certainty grading.

All extracted data, effect-size selection decisions, model specifications, and analysis materials were documented to support reproducibility. The Supplementary Materials provide the extracted-estimate tables, effect-size selection information, risk-of-bias judgments, and sensitivity and influence diagnostics supporting the primary analyses.

## Figures and Tables

**Table 1:** Primary human epidemiological study–outcome estimates included in the outcome-specific quantitative synthesis.

Study	Country/Region	Design	Population/Sample	Exposure Matrix	Analytical Method	Outcome Group	Selected Adjusted Estimate	Key Covariates	RoB	Synthesis Role
Li et al. (2011) [21]	China, Three Gorges Reservoir region	Cross-sectional	Children, n = 1322	Environmental MC exposure/drinking-water-related exposure	Environmental/drinking-water exposure classification; no individual serum assay	Hepatic	Liver damage: OR 1.72 (95% CI 1.05–2.76)	HBV, hepatotoxic drugs, BMI, physical activity, passive smoking	Moderate	Primary hepatic evidence
Zheng et al. (2017) [22]	China, Southwest	Population-based case–control	*HCC cases and controls, n = 428*	Serum MC-LR	Serum MC-LR measured by ELISA	Hepatic	HCC, high vs. low MC-LR: OR 2.90 (95% CI 1.50–5.50)	Sex, smoking, alcohol, liver disease history, family history, aflatoxin, HBV	Low–moderate	Primary hepatic evidence; quartile and interaction estimates in Table S5
Gao et al. (2023) [24]	China, Hunan	Case–control	*CKD cases and controls, n = 270*	Serum/plasma MCs	ELISA/ICP-MS for co-exposures	Renal	CKD, highest MC category vs. reference: OR 4.81 (95% CI 1.96–11.81)	Hypertension, arsenic exposure, other CKD covariates	Low–moderate	Primary renal evidence; arsenic co-exposure estimates in Table S5
Feng et al. (2023) [25]	China, Hunan	Cross-sectional	*Adult population, n = 720*	Serum MCs	ELISA	Metabolic	Dyslipidemia, Q4 vs. Q1 MCs: OR 2.92 (95% CI 1.80–4.73)	Lipid-related metals, including Zn, Se, Fe, Ti, Mo, Cd	Low–moderate	Primary metabolic evidence
Feng et al. (2024b) [27]	China, Central China	Cross-sectional	*Adults, n = 591*	Serum MCs	Serum MCs measured by ELISA (LOD 0.01 μg/L)	Metabolic	Metabolic syndrome, Q4 vs. Q1 serum MCs: OR 2.10 (95% CI 1.19–3.70)	Age, sex, smoking, alcohol consumption, BMI, physical activity, income, education, hypertension, diabetes, and other reported covariates as available in the original model	Moderate	Primary metabolic evidence
Lin et al. (2023) [26]	China	Nested case–control	*Pregnant women, n = 357*	Serum MC-LR	Biomonitoring-based serum MC assessment	Metabolic	Gestational diabetes: OR 1.92 (95% CI 1.09–3.39)	Age, BMI, gestational weight gain, education, income, family history	Moderate	Primary metabolic evidence
Zeng et al. (2017) [36]	China, Southwest China/Three Gorges Reservoir region	Cross-sectional	*Adults, n = 5493*	Estimated dietary/environmental MC-LR exposure	Exposure estimated from environmental/dietary MC-LR exposure data; logistic regression	Metabolic	Metabolic syndrome; higher vs. lower MC-LR exposure: OR 0.89 (95% CI 0.77–1.02)	Age, sex, abnormal liver function, HBV infection, aflatoxin B1 exposure, alcohol drinking, cigarette smoking, labor strength, education, family income, and dietary factors	Moderate	Primary metabolic evidence; null/non-significant estimate retained to reduce selective emphasis on positive associations

Note. MC, microcystin; OR, odds ratio; CI, confidence interval; ELISA, enzyme-linked immunosorbent assay; ICP-MS, inductively coupled plasma mass spectrometry; MALDI-TOF MS, matrix-assisted laser desorption/ionization time-of-flight mass spectrometry; RoB, risk of bias; HBV, hepatitis B virus; HCC, hepatocellular carcinoma; CKD, chronic kidney disease. Primary estimates were selected according to the most fully adjusted main population-level estimate for each study–outcome group. Dose–response, subgroup, interaction, co-exposure, mediation, sensitivity, review-derived, surveillance, and contextual estimates were extracted separately and are reported in Supplementary Table S5. Co-exposure estimates, including MC–cadmium and MC–arsenic contrasts, were interpreted as secondary effect-modification evidence and were not treated as independent primary observations. The MC–cadmium CKD estimate reported by Feng et al. (2022) [23] was retained in Supplementary Table S5 as secondary co-exposure evidence but was not included in the primary renal model.

**Table 2:** Outcome-specific meta-analyses and certainty assessment for chronic microcystin exposure and human health outcomes.

Model/Outcome Group	Studies	Estimates	Summary OR	95% CI	I^2^	τ^2^	Prediction Interval	Model Status	Main RoB Concern	Certainty	Interpretation
Hepatic outcomes	2	2	2.13	1.29–3.53	37%	0.10	0.84–5.40	Primary	Confounding/exposure assessment	Low	Relatively consistent but limited to two studies with different hepatic endpoints
Renal outcomes	1	1	4.81	1.96–11.81	NA	NA	NA	Primary descriptive single-estimate summary	Co-exposure/confounding	Low	Positive CKD signal; mixture-aware interpretation required
Metabolic outcomes	4	4	1.73	1.00–2.98	91%	0.26	0.56–5.36	Primary	Outcome heterogeneity/exposure assessment	Low	Suggestive but heterogeneous evidence

Note. OR, odds ratio; CI, confidence interval; RoB, risk of bias; NA, not applicable. The primary interpretation should be based on outcome-specific models and the one-study–one-main-estimate rule. The expanded extracted-estimate comparison is reported for transparency and comparison with the broader extracted estimate set. It should not be interpreted as the main pooled effect because some parent studies contributed multiple statistically dependent estimates.

**Table 3:** Toxicological evidence domains, major uncertainties, and research priorities for chronic microcystin exposure assessment.

Toxicological Domain	Role in Synthesis	Evidence Pattern	Main Limitation	Priority for Interpretation and Future Research
Mechanistic toxicology	Biological plausibility	OATP-mediated uptake, PP1/PP2A inhibition, oxidative stress, mitochondrial dysfunction, inflammatory signaling, and cytoskeletal injury.	Translation from experimental systems to chronic, low-dose, multi-route human exposure remains uncertain.	Use mechanistic evidence to assess coherence, not as a substitute for human epidemiological evidence.
Exposure assessment	Source of heterogeneity	Environmental proxies, water and dietary measurements, and internal biomarkers were used across studies.	Exposure matrices, windows, thresholds, and analytical specificity were inconsistent.	Harmonize exposure windows and link external exposure measurements with internal dose.
Biomonitoring	Internal-dose characterization	Serum, plasma, and urine measurements provide more direct evidence of absorbed microcystins.	Single measurements may not represent chronic exposure; assay specificity varies.	Use repeated sampling and validated methods with transparent LOD/LOQ and quality-control reporting.
Congener-specific analysis	Analytical confirmation	Congener-specific confirmation can distinguish MC-LR and other variants from cross-reactive or total-toxin signals.	ELISA and non-targeted classifications may introduce cross-reactivity and exposure misclassification.	Confirm congeners using LC-MS/MS or equivalent validated analytical methods.
Co-exposure and mixture toxicity	Effect modification	Associations may be modified by arsenic, cadmium, aflatoxin, hepatitis infection, alcohol, and other stressors.	Few studies were designed to separate independent and joint effects.	Apply mixture-aware models and report both main effects and interaction contrasts.
Prospective epidemiology	Causal inference	Existing evidence is dominated by cross-sectional and case–control designs.	Temporality, residual confounding, and geographic concentration limit certainty.	Conduct prospective multi-region cohorts with repeated biomonitoring and organ-specific clinical endpoints.

Note. MC, microcystin; CyanoHAB, cyanobacterial harmful algal bloom; OATP, organic anion-transporting polypeptide; PP1/PP2A, protein phosphatases 1 and 2A; LC–MS/MS, liquid chromatography–tandem mass spectrometry. Contextual domains were used to interpret heterogeneity, biological plausibility, implementation gaps, and research priorities. These domains were used for interpretation and were not included as independent effect estimates in the primary quantitative synthesis. Mechanistic toxicology was considered only as supporting evidence for biological coherence and was not statistically pooled with human epidemiological estimates or assigned a separate certainty rating.

## Data Availability

No new data were created or analyzed in this study. Data sharing is not applicable to this article.

## Supplementary Materials

The following supporting information can be downloaded at: https://www.mdpi.com/article/10.3390/toxins18090373/s1. The supporting information includes Supplementary Tables S1–S7 and Supplementary Figures S1–S3, comprising the evidence-domain map, database-adapted search strategy, extracted epidemiological estimates, risk-of-bias assessment, guideline values, sensitivity diagnostics, and the directed acyclic graph. Supplementary File: PRISMA 2020 Checklist and PRISMA 2020 for Abstracts Checklist.

## Abbreviations

The following abbreviations are used in this manuscript:

AOP	Adverse Outcome Pathway
CI	Confidence interval
CKD	Chronic kidney disease
CyanoHAB	Cyanobacterial harmful algal bloom
ELISA	Enzyme-linked immunosorbent assay
HCC	Hepatocellular carcinoma
LC-MS/MS	Liquid chromatography-tandem mass spectrometry
MC	Microcystin
OATP	Organic anion-transporting polypeptide
OR	Odds ratio
PECO	Population-Exposure-Comparator-Outcome
PP1/PP2A	Protein phosphatases 1 and 2A
PRISMA	Preferred Reporting Items for Systematic Reviews and Meta-Analyses
RoB	Risk of bias
