# Supplementary material for: Human Health Effects of Chronic Microcystin Exposure: A Systematic Review and Meta-Analysis of Hepatic, Renal, and Metabolic Outcomes
Source: Toxins (Basel). 2026 Aug 30;18(9):373. doi: 10.3390/toxins18090373 (PMC13611463; doi:10.3390/toxins18090373)
Supplement: Supplementary file 1 [file toxins-18-00373-s001.zip › toxins-4516077_Supplementary_Materials_1.pdf]

Supplementary Materials: Human Health Effects of Chronic Microcystin Exposure: A Systematic Review and Meta-Analysis of Hepatic , Renal, and Metabolic Outcomes

Seong-Dae Moon, Dae-Sik Hwang, Jung-Suk Lee, Su-Ok Hwang, Hans W. Paerl and Baik-Ho Kim

Contents

Supplementary Table S1. Contextual evidence domains used for evidence mapping.

Supplementary Table S2. Search concepts, executed search strings, and evidence-domain mapping used for PubMed, Scopus, and Web of Science searches.

Supplementary Table S3. Thematic distribution of screened records and synthesized studies across contextual evidence domains.

Supplementary Table S4. Standardized data-extraction template for included studies.

Supplementary Table S5. Extracted epidemiological effect estimates for chronic microcystin exposure and hepatic, renal, and metabolic outcomes.

Supplementary Table S6. Risk-of-bias assessment for primary epidemiological estimates.

Supplementary Table S7. International guideline values for microcystins in drinking and recreational waters.

Supplementary Figure S1. Evidence-density heatmap by evidence domain and bibliographic database.

Supplementary Figure S2. Sensitivity and influence diagnostics for the expanded extracted-estimate comparison.

Supplementary Figure S3. Integrated directed acyclic graph for chronic microcystin exposure and hepatic, renal, and metabolic outcomes.

Supplementary Table S1. Contextual evidence domains used for evidence mapping
This table summarizes the evidence domains used to organize human epidemiology, mechanistic toxicology, environmental exposure, and supporting risk-context literature. Only eligible adjusted human epidemiological estimates were included in the primary quantitative synthesis; mechanistic and contextual evidence was used to evaluate toxicological coherence and exposure plausibility.

Evidence domain	Domain focus	Role in this review
P1. Epidemiology and public-health evidence	Human disease outcomes; OR/RR/HR estimates; hepatic, renal, and metabolic endpoints	Identify and synthesize human epidemiological evidence eligible for qualitative review and quantitative synthesis.
P2. Mechanistic and translational toxicology	OATP-mediated uptake; PP1/PP2A inhibition; oxidative stress; mitochondrial injury; inflammatory signaling	Support biological plausibility and interpretation of organ-specific pathways without replacing human epidemiological evidence.

P3. Economic and societal context	Cost-of-illness, productivity loss, willingness-to-pay, treatment and mitigation costs	Provide exploratory public-health context for potential socioeconomic burden; not used for primary causal inference, meta-analysis weighting, or certainty grading.
P4. Governance, monitoring, and policy systems	Regulatory standards, surveillance capacity, early-warning systems, water-safety planning, treatment readiness	Identify implementation gaps and surveillance needs relevant to exposure prevention and risk translation.
P5. Environmental drivers and exposure pathways	Nutrient loading, climate forcing, hydrology, bloom dynamics, aerosolization, aquatic-food exposure	Characterize environmental conditions and exposure pathways that may influence chronic or repeated MC exposure.
P6. Vulnerability, equity, and implementation context	Infrastructure gaps, water insecurity, monitoring disparities, vulnerable populations	Identify contextual vulnerability and research gaps relevant to targeted surveillance and exposure-prevention strategies

Note. P1 provided the basis for the primary human epidemiological synthesis. P2–P6 were used as supporting or contextual evidence domains and were not statistically pooled with the primary adjusted human epidemiological estimates.

Supplementary Table S2. Search concepts, executed search strings, and evidence-domain mapping used for PubMed, Scopus, and Web of Science searches.

Database-specific search strategies are summarized below. Because the original database export histories were not retained in a form that allowed verbatim reconstruction of every executed query, the searches are reported as a retrospectively reconstructed, database-adapted strategy based on the documented PECO concepts, retained search terms, date limits, and evidence-domain mapping. The reconstruction is intended to make the search logic transparent without implying that the strings below are exact system-generated export logs.

**Database**	**Search timing and coverage**	**Evidence-domain mapped records in Fig. S1**	**Interpretation**
PubMed	Initial search completed to 1 Nov 2025; update search conducted in Apr 2026; publication coverage 1 Jan 1990–1 Nov 2025	2,461 (P1–P6 cell counts summed)	Domain-mapped count; not the raw database total used in the PRISMA identification box
Scopus	Initial search completed to 1 Nov 2025; update search conducted in Apr 2026; publication coverage 1 Jan 1990–1 Nov 2025	1,570 (P1–P6 cell counts summed)	Domain-mapped count; not the raw database total used in the PRISMA identification box
Web of Science Core Collection	Initial search completed to 1 Nov 2025; update search conducted in Apr 2026; publication coverage 1 Jan 1990–1 Nov 2025	1,484 (P1–P6 cell counts summed)	Domain-mapped count; not the raw database total used in the PRISMA identification box

PubMed (reconstructed search syntax): ((microcystin*[Title/Abstract] OR "microcystin-LR"[Title/Abstract] OR cyanotoxin*[Title/Abstract] OR "cyanobacterial harmful algal bloom*"[Title/Abstract] OR CyanoHAB*[Title/Abstract]) AND (human*[Title/Abstract] OR epidemiolog*[Title/Abstract] OR cohort[Title/Abstract] OR "case-control"[Title/Abstract] OR "cross-sectional"[Title/Abstract] OR biomonitor*[Title/Abstract]) AND (hepatic[Title/Abstract] OR liver[Title/Abstract] OR "hepatocellular carcinoma"[Title/Abstract] OR renal[Title/Abstract] OR kidney[Title/Abstract] OR "chronic kidney

disease"[Title/Abstract] OR dyslipid*[Title/Abstract] OR "metabolic syndrome"[Title/Abstract] OR diabetes[Title/Abstract])) AND ("1990/01/01"[Date - Publication] : "2025/11/01"[Date - Publication]).

Scopus (reconstructed search syntax): TITLE-ABS-KEY((microcystin* OR "microcystin-LR" OR cyanotoxin* OR "cyanobacterial harmful algal bloom*" OR CyanoHAB*) AND (human* OR epidemiolog* OR cohort OR "case-control" OR "cross-sectional" OR biomonitor*) AND (hepatic OR liver OR "hepatocellular carcinoma" OR renal OR kidney OR "chronic kidney disease" OR dyslipid* OR "metabolic syndrome" OR diabetes)) AND PUBYEAR > 1989 AND PUBYEAR < 2026.

Web of Science Core Collection (reconstructed search syntax): TS=((microcystin* OR "microcystin-LR" OR cyanotoxin* OR "cyanobacterial harmful algal bloom*" OR CyanoHAB*) AND (human* OR epidemiolog* OR cohort OR "case-control" OR "cross-sectional" OR biomonitor*) AND (hepatic OR liver OR "hepatocellular carcinoma" OR renal OR kidney OR "chronic kidney disease" OR dyslipid* OR "metabolic syndrome" OR diabetes)) AND PY=(1990-2025).

Search-count note. The heatmap in Supplementary Fig. S1 reports evidence-domain mapped counts (PubMed: 2,461; Scopus: 1,570; Web of Science: 1,484). These values summarize records assigned to the P1–P6 mapping matrix and should not be interpreted as the raw, mutually exclusive database totals underlying the 9,021 records in the PRISMA identification stage. The original exact database-level raw counts were not recoverable from the retained files; therefore, no unsupported database-specific raw totals were imputed. The April 2026 update identified no additional independent adjusted human epidemiological estimate eligible for the primary quantitative synthesis.

Evidence domain	Domain / focus	Documented full Boolean search string	Executed search string / noise-reduced version	Purpose / notes
P1. Epidemiology and public-health evidence	Human health risk, OR/RR/HR estimates, disease burden, and hepatic, renal, or metabolic outcomes	(epidemiology OR "human health" OR hospitalization OR incidence OR prevalence OR "case-control" OR "cohort study" OR "time-series" OR "risk ratio" OR "odds ratio" OR "hazard ratio" OR DALY* OR "disease burden") AND Humans	(microcystin AND (epidemiology OR "odds ratio" OR "hazard ratio" OR hospitalization)) NOT (mouse OR rat OR zebrafish)	Removed animal-only studies and prioritized human epidemiological evidence for hepatic, renal, and metabolic outcomes, including hepatocellular carcinoma, chronic kidney disease, dyslipidemia, metabolic syndrome, gestational diabetes, and related clinical or biomarker endpoints.
P2. Mechanistic and translational toxicology	OATP-mediated uptake, PP1/PP2A inhibition, oxidative stress, mitochondrial injury, inflammatory signaling	(toxicity OR hepatotoxicity OR nephrotoxicity OR "oxidative stress" OR apoptosis OR PP1 OR PP2A OR OATP OR ABCB1 OR GST OR AOP OR toxicokinetics OR "mitochondrial dysfunction")	(microcystin AND (oxidative OR hepatotoxicity OR nephrotoxicity OR PP1 OR PP2A OR OATP OR mitochondrial)) NOT (wastewater OR sludge OR bioremediation)	Excluded environmental-engineering noise and retained mechanistic studies relevant to biological plausibility. These studies supported interpretation of OATP-mediated uptake, PP1/PP2A inhibition, oxidative stress, mitochondrial dysfunction, and organ-specific injury, but were not included in the primary quantitative synthesis.

P3. Economic and societal context	Cost of illness, productivity loss, willingness-to-pay, treatment cost, mitigation cost	("economic cost" OR "cost-of-illness" OR "healthcare expenditure" OR "medical cost" OR "productivity loss" OR WTP OR "recreation value" OR "treatment cost")	(microcystin AND (cost OR economic OR WTP OR "treatment cost" OR mitigation))	Used only to identify contextual public-health and economic evidence. These records were not used for primary causal inference, meta-analysis, or certainty grading, and no separate economic model was retained in the final synthesis.
P4. Governance, monitoring, and policy systems	Regulation, monitoring, water-safety planning, surveillance capacity, remote sensing, early warning	(policy OR governance OR regulation OR "water safety plan" OR monitoring OR "remote sensing" OR satellite OR "machine learning" OR AI OR "risk management")	(microcystin AND (monitoring OR governance OR "remote sensing" OR satellite OR "risk management" OR "water safety plan"))	Retained freshwater monitoring, governance, treatment-readiness, and surveillance studies relevant to exposure prevention and risk translation. These studies were treated as contextual evidence.
P5. Environmental drivers and exposure pathways	Nutrient loading, hydrology, climate forcing, bloom dynamics, aerosol exposure, food-web exposure	(environment* OR eutrophication OR "nutrient loading" OR nitrogen OR hydrology OR "cyanobacterial bloom" OR aerosol* OR "airborne microcystin" OR "food contamination")	(microcystin AND (eutrophication OR nutrient OR hydrology OR bloom OR aerosol OR food)) NOT (wastewater OR sludge OR soil)	Removed overly broad environmental terms while retaining studies relevant to environmental drivers, bloom formation, and multi-route exposure pathways. Agriculture-related records were not excluded when relevant to catchment nutrient loading.
P6. Vulnerability, equity, and implementation context	Water insecurity, infrastructure gaps, monitoring disparities, vulnerable populations	("environmental justice" OR "health equity" OR disparity OR "vulnerable population" OR "water insecurity" OR "infrastructure gap" OR "implementation capacity")	(microcystin AND (equity OR justice OR vulnerable OR "water insecurity" OR "infrastructure gap" OR monitoring disparity))	Used to identify contextual vulnerability and implementation-related evidence. These records informed evidence-gap mapping and surveillance priorities, but were not treated as primary causal evidence.

Note. Microcystin was used as the primary sentinel cyanotoxin because it has the most developed human epidemiological, toxicological, and regulatory evidence base relevant to this review. Searches covered records published from January 1, 1990, to November 1, 2025. A final update search was conducted in April 2026 using the same PECO-based concepts and did not identify any additional independent adjusted human epidemiological estimate eligible for the primary quantitative synthesis. P1–P6 domain strings were adapted to the field syntax of each database; mechanistic, environmental, economic, governance, and vulnerability records were used only as supporting or contextual evidence unless they met the primary PECO criteria.

Supplementary Table S3. Thematic distribution of screened records and synthesized studies across contextual evidence domains.

This table distinguishes the 34 human epidemiological studies addressing the primary review question from 158 supporting studies retained only for mechanistic or contextual interpretation. The seven independent estimates used in the primary quantitative synthesis were selected from the 34 human studies. Supporting studies were not pooled with human epidemiological evidence.

An itemized list of the 1,266 full-text articles excluded after assessment is not available in the retained review files. Aggregate exclusion reasons were unrelated MC exposure, ineligible evidence domain or outcome, and insufficient information for qualitative or quantitative synthesis. No study-level exclusion reasons were reconstructed retrospectively; this is acknowledged as a review-process limitation in the manuscript.

Evidence domain	Full-text records assessed or mapped (N = 1,458)	Final studies retained in synthesis (N = 192)	Main contribution to this review
P1. Epidemiology and public-health evidence	48	34	Identified human epidemiological evidence relevant to chronic or repeated MC exposure and hepatic, renal, or metabolic outcomes.
P2. Mechanistic and translational toxicology	1,193	86	Supported biological plausibility through OATP-mediated uptake, PP1/PP2A inhibition, oxidative stress, mitochondrial dysfunction, inflammatory signaling, and organ-specific injury pathways.
P3. Economic and societal context	5	6	Provided exploratory public-health context on potential cost-of-illness, productivity loss, treatment, mitigation, and affordability implications.
P4. Governance, monitoring, and policy systems	60	6	Informed interpretation of monitoring capacity, guideline implementation, treatment readiness, early-warning systems, and risk-translation needs.
P5. Environmental drivers and exposure pathways	146	54	Characterized nutrient loading, hydrology, climate forcing, bloom dynamics, and multi-route exposure opportunities relevant to chronic or repeated MC exposure.
P6. Vulnerability, equity, and implementation context	6	6	Identified contextual vulnerability, infrastructure gaps, water insecurity, and monitoring disparities relevant to future surveillance and exposure prevention.

Note. Domain labels were used only for evidence mapping and interpretation. P1 provided the basis for the primary human epidemiological synthesis, whereas P2–P6 were used as supporting or contextual evidence unless individual studies met the PECO criteria for primary quantitative synthesis. Final retained studies may include records identified through reference-list screening or supplementary targeted searches; therefore, domain-specific final counts may occasionally exceed the initial mapped full-text count for a domain. Economic, governance, environmental, and vulnerability-related studies were not used as primary causal evidence or statistically pooled with the adjusted human epidemiological estimates. For P3, one additional economic-context record was identified during reference-list or targeted supplementary searching after the initial domain mapping; therefore, the retained P3 count exceeds the initially mapped full-text count.

Supplementary Table S4. Standardized data-extraction template for included studies.

Data fields were used to extract information from eligible human epidemiological studies and, where relevant, to map mechanistic toxicology, exposure pathways, analytical methods, and contextual evidence. Only adjusted human epidemiological estimates meeting the PECO criteria were eligible for the primary quantitative synthesis.

Domain	Variable	Definition / coding rule
Study identification	Study ID	First author and year, e.g., Feng et al. (2023)
	Full citation	Full bibliographic citation, including title, journal, publication year, and DOI where available
	Country / region	Country, study region, and, where relevant, WHO region or World Bank income classification
Study design	Study type	Cohort, case-control, nested case-control, cross-sectional, panel, time-series, ecological, biomonitoring, mechanistic, or contextual study design
	Study period	Start and end years; bloom-season coverage where applicable
Population characteristics	Sample size	Number of participants, cases/controls, person-years, or environmental samples, as applicable
	Age distribution	Mean, median, range, or predefined age categories
	Sex distribution	Percentage or number of female and male participants where reported
	Vulnerable or susceptible subgroups	Children, older adults, pregnant populations, individuals with CKD, liver disease, metabolic syndrome, occupational or recrea tional exposure, or other reported susceptible groups
	Socioeconomic variables	Income, education, occupation, rural/urban status, household water source, sanitation, or water-insecurity indicators where reported
Exposure assessment	Exposure matrix	Drinking water, raw water, surface water, aquatic food, recreational water, aerosol, serum, plasma, urine, or other reported mat rix

	Analytical method	ELISA, LC–MS/MS, HRMS, MALDI-TOF MS, ICP-MS for co-exposures, or other reported analytical method; LOD/LOQ and calibration information where available
	Exposure definition	MC concentration, exposure category, exposure tier, biomonitoring measure, environmental proxy, or modeled exposure estim ate
	Exposure window / sampling frequency	Sampling count, temporal resolution, exposure period, chronic or repeated exposure definition, and bloom-season coverage where reported
	Multi-route exposure	Drinking water, aquatic food, recreational contact, aerosol exposure, or other routes; coded as reported by each study
Environmental context	Nutrient and bloom indicators	TN, TP, DIN, DIP, chlorophyll-a, phycocyanin, cyanobacterial biomass, Cyanobacteria Index, or other bloom indicators where available
	Hydrologic factors	Residence time, retention time, flow, water level, stratification, mixing regime, or hydrologic alteration
	Meteorological and climate factors	Temperature, precipitation, wind, extreme events, seasonality, climate anomalies, or long-term warming indicators
Outcome assessment	Outcome type	Hepatic, renal, or metabolic endpoints, including hepatocellular carcinoma, chronic kidney disease, dyslipidemia, metabolic syndrome, gestational diabetes, liver injury markers, renal injury markers, and lipid-profile abnormalities.
	Outcome measurement	Clinical diagnosis, ICD code, registry record, medical record, biomarker, hospitalization, mortality, or other reported outcome measure
	Outcome ascertainment	Registry, electronic medical record, medical chart, survey, clinical examination, laboratory biomarker, or active surveillance s ystem
Effect estimates	Main effect estimate	OR, RR, HR, IRR, beta coefficient, or other reported effect estimate with 95% CI where available

	Adjusted covariates	Age, sex, socioeconomic status, HBV/HCV, aflatoxin, alcohol, smoking, BMI, hypertension, diabetes, pregnancy-related variables, metals, pesticides, seasonality, meteorology, or other study-specific covariates
	Estimate role	Primary main estimate, dose-response estimate, subgroup estimate, interaction estimate, co-exposure estimate, mediation estimate, sensitivity estimate, review-derived estimate, surveillance estimate, or contextual estimate
	Dose-response information	Exposure categories, trend tests, spline models, monotonicity, or threshold information where reported
Mechanistic and biomarker information	Internal dose or biomarker	Serum, plasma, or urine MCs; MC-LR; MC-GSH; MC-Cys; oxidative stress markers; inflammatory markers; renal, hepatic, or metabolic biomarkers where reported
	Mechanistic linkage	OATP-mediated uptake, PP1/PP2A inhibition, oxidative stress, mitochondrial dysfunction, inflammatory signaling, cytoskeletal disruption, fibrosis, tubular injury, or metabolic disturbance
Contextual economic information	Cost or affordability variables	Cost-of-illness, treatment cost, mitigation cost, productivity loss, willingness-to-pay, PPP adjustment, or affordability indicator where reported
Governance and vulnerability context	Monitoring and treatment capacity	Monitoring frequency, toxin-confirmation capacity, LC–MS/MS availability, remote sensing coverage, water-treatment capacity, advisory systems, or emergency response capacity
	Vulnerability indicators	Water insecurity, infrastructure gaps, rural/urban disparities, environmental-justice markers, limited monitoring capacity, or vulnerable populations
Risk of bias	Domains assessed	Participant selection, exposure assessment, outcome assessment, confounding, temporality, missing data, selective reporting, and statistical analysis

Overall judgment	Low, low–moderate, moderate, serious/high concern, or not applicable, assigned at the study–outcome estimate level where relevant
Special comments	Co-exposures, missing data, non-independence of estimates, subgroup structure, sensitivity-analysis status, review-derived estimates, or study-specific limitations

Note. Variables related to environmental drivers, mechanistic toxicology, economics, governance, and vulnerability were used for contextual interpretation and evidence mapping. They were not statistically pooled with the primary adjusted human epidemiological estimates unless the study met the PECO eligibility criteria for inclusion in the primary quantitative synthesis.

Supplementary Table S5. Extracted epidemiological effect estimates for chronic microcystin exposure and hepatic, renal, and metabolic outcomes.

This table provides the full set of extracted effect estimates from human epidemiological studies, including primary main estimates, dose-response estimates, subgroup analyses, co-exposure interactions, mediation analyses, sensitivity estimates, and estimates excluded from the primary meta-analysis. Estimates are classified according to their role in the synthesis to distinguish primary study–outcome estimates from secondary or contextual evidence.

Part A. Primary main estimates

**Study**	**Outcome group**	**Specific outcome**	**Outcome definition / ICD code, if reported**	**N**	**Exposure matrix**	**Analytical method**	**Estimate type**	**Effect metric**	**Adjusted estimate**	**Key covariates**	**Synthesis role**	**Notes**
Li et al. (2011)	Hepatic	Liver damage in children; MC-exposed vs unexposed	Biomarker-defined liver damage; ICD code not reported	1,322	Environmental/drinking-water-related exposure	Environmental exposure classification; clinical biochemistry	Primary main estimate	OR	1.72 (95% CI 1.05–2.76)	HBV, hepatotoxic drugs, BMI, physical activity, passive smoking	Primary hepatic evidence	Selected as a population-level hepatic estimate.

Zheng et al. (2017)	Hepatic	Hepatocellular carcinoma; high vs low serum MC-LR	Hepatocellular carcinoma; ICD code reported only if used in source	428	Serum MC-LR	Biomonitoring-based serum MC assessment	Primary main estimate	OR	2.90 (95% CI 1.50–5.50)	Sex, smoking, alcohol, liver disease history, family history, aflatoxin, HBV	Primary hepatic evidence	Dose-response and interaction estimates are listed in Parts B and C.
Gao et al. (2023)	Renal	Chronic kidney disease; highest MC category vs reference	Chronic kidney disease; clinical/laboratory-defined CKD; ICD code not reported unless used in source	270	Serum/plasma MCs	ELISA; ICP-MS for arsenic co-exposure	Primary main estimate	OR	4.81 (95% CI 1.96–11.81)	Hypertension, arsenic concentration	Primary renal evidence	Selected as highest MC category; arsenic joint-effect estimates are listed in Part C.
Feng et al. (2023)	Metabolic	Dyslipidemia; Q4 vs Q1 serum MCs	Dyslipidemia; lipid-profile-defined outcome; ICD code not reported	720	Serum MCs	ELISA	Primary main estimate	OR	2.92 (95% CI 1.80–4.73)	Lipid-related metals (Zn, Se, Fe, Ti, Mo, Cd)	Primary metabolic evidence	Specific lipid endpoints and subgroup estimates are listed in Parts B and C.

Feng et al. (2024b)	Metabolic	Metabolic syndrome; Q4 vs Q1 serum MCs or MC-LR exposure	Metabolic syndrome; study-defined diagnostic criteria; ICD code not reported	591	Serum MCs / biomonitoring-based MC exposure	Biomonitoring-based serum MC assessment; logistic regression	Primary main estimate	OR	2.10 (95% CI 1.19–3.70)	Age, sex, smoking, alcohol consumption, BMI, physical activity, income, education, hypertension, diabetes, and other reported covariates as available in the original model	Primary metabolic evidence	Mediation or inflammatory-marker estimates, if reported, are listed in Part C and were not used as independent primary estimates.
Lin et al. (2023)	Metabolic	Gestational diabetes; high vs low serum MC-LR	Gestational diabetes mellitus; clinical or pregnancy-screening definition; ICD code not reported	357	Serum MC-LR	Biomonitoring-based serum MC assessment	Primary main estimate	OR	1.92 (95% CI 1.09–3.39)	Age, BMI, gestational weight gain, education, income, family history	Primary metabolic evidence	Subgroup estimates are listed in Part C.
Zeng et al. (2017)	Metabolic	Metabolic syndrome; higher vs lower MC-LR exposure	Metabolic syndrome; study-defined diagnostic criteria; ICD code not reported	5,493	Estimated dietary/environmental MC-LR exposure	Exposure estimated from environmental/dietary MC-LR data; logistic regression	Primary main estimate	OR	0.89 (95% CI 0.77–1.02)	Age, sex, abnormal liver function, HBV infection, aflatoxin B1 exposure, alcohol drinking, cigarette smoking, labor strength, education, family income, and dietary factors	Primary metabolic evidence; null/non-significant estimate	Retained to avoid selective emphasis on positive associations and to preserve non-significant metabolic evidence.

Part B. Dose-response and outcome-specific secondary estimates

Study	Outcome group	Specific outcome	Outcome definition / ICD code, if reported	N	Exposure matrix	Analytical method	Estimate type	Effect metric	Adjusted estimate	Key covariates	Synthesis role	Notes
Zheng et al. (2017)	Hepatic	HCC; serum MC-LR quartile 2 vs quartile 1	HCC	428	Serum MC-LR	Biomonitoring-based serum MC assessment	Dose-response estimate	OR	1.30 (95% CI 0.50–3.10)	Major HCC confounders adjusted in parent study	Secondary dose-response evidence	Not treated as an independent primary estimate.
Zheng et al. (2017)	Hepatic	HCC; serum MC-LR quartile 3 vs quartile 1	HCC	428	Serum MC-LR	Biomonitoring-based serum MC assessment	Dose-response estimate	OR	2.60 (95% CI 1.10–6.50)	Major HCC confounders adjusted in parent study	Secondary dose-response evidence	Supports monotonic exposure-response interpretation.
Zheng et al. (2017)	Hepatic	HCC; serum MC-LR quartile 4 vs quartile 1	HCC	428	Serum MC-LR	Biomonitoring-based serum MC assessment	Dose-response estimate	OR	4.00 (95% CI 1.60–9.90)	Major HCC confounders adjusted in parent study	Secondary dose-response evidence	Highest quartile estimate; not counted separately from the primary study estimate.
Gao et al. (2023)	Renal	CKD; MCs Q2 vs Q1	CKD	270	Serum/plasma MCs	ELISA; ICP-MS for arsenic	Dose-response estimate	OR	3.70 (95% CI 1.38–9.93)	Hypertension and arsenic concentration	Secondary dose-response evidence	Not treated as an independent primary estimate.
Gao et al. (2023)	Renal	CKD; MCs Q3 vs Q1	CKD	270	Serum/plasma MCs	ELISA; ICP-MS for arsenic	Dose-response estimate	OR	4.20 (95% CI 1.62–10.90)	Hypertension and arsenic concentration	Secondary dose-response evidence	Not treated as an independent primary estimate.

Study	Outcome group	Specific outcome	Outcome definition / ICD code, if reported	N	Exposure matrix	Analytical method	Estimate type	Effect metric	Adjusted estimate	Key covariates	Synthesis role	Notes
Gao et al. (2023)	Renal	CKD; MCs Q4 (≥0.19 μg/L) vs Q1	CKD	270	Serum/plasma MCs	ELISA; ICP-MS for arsenic	Dose-response estimate	OR	4.81 (95% CI 1.96–11.81)	Hypertension and arsenic concentration	Primary selected estimate and dose-response evidence	If selected for primary model, do not count again in dose-response pooling.
Feng et al. (2023)	Metabolic	HyperLDL-C; Q4 vs Q1 MCs	High LDL cholesterol	720	Serum MCs	ELISA	Outcome-specific estimate	OR	1.74 (95% CI 0.76–3.96)	Lipid-related metals	Secondary metabolic evidence	Non-significant endpoint-specific estimate.
Feng et al. (2023)	Metabolic	HyperTC; Q4 vs Q1 MCs	High total cholesterol	720	Serum MCs	ELISA	Outcome-specific estimate	OR	1.76 (95% CI 0.83–3.70)	Lipid-related metals	Secondary metabolic evidence	Non-significant endpoint-specific estimate.
Feng et al. (2023)	Metabolic	HyperTG; Q4 vs Q1 MCs	High triglycerides	720	Serum MCs	ELISA	Outcome-specific estimate	OR	4.25 (95% CI 2.40–7.52)	Lipid-related metals	Secondary metabolic evidence	Endpoint-specific estimate; do not count independently in primary model if dyslipidemia main estimate is selected.
Feng et al. (2023)	Metabolic	HypoHDL-C; Q4 vs Q1 MCs	Low HDL cholesterol	720	Serum MCs	ELISA	Outcome-specific estimate	OR	2.45 (95% CI 1.01–5.95)	Lipid-related metals	Secondary metabolic evidence	Endpoint-specific estimate.

Part C. Subgroup, interaction, co-exposure, and mediation estimates

Study	Outcome group	Specific outcome	Outcome definition / ICD code, if reported	N	Exposure matrix	Analytical method	Estimate type	Effect metric	Adjusted estimate	Key covariates	Synthesis role	Notes
Zheng et al. (2017)	Hepatic	HCC; female subgroup, high vs low MC-LR	HCC	62	Serum MC-LR	Biomonitoring-based serum MC assessment	Subgroup estimate	OR	0.60 (95% CI 0.10–4.70)	Subgroup analysis	Effect-modification evidence	Not statistically significant; not used as primary estimate.
Zheng et al. (2017)	Hepatic	HCC; male subgroup, high vs low MC-LR	HCC	366	Serum MC-LR	Biomonitoring-based serum MC assessment	Subgroup estimate	OR	3.30 (95% CI 1.60–6.90)	Subgroup analysis	Effect-modification evidence	Sex-stratified estimate; dependent on parent study.
Zheng et al. (2017)	Hepatic	HCC; high MC-LR and alcohol drinking	HCC	428	Serum MC-LR; alcohol co-exposure	Biomonitoring-based serum MC assessment	Interaction estimate	OR	6.80 (95% CI 2.70–17.20)	Interaction: alcohol and MC-LR	Effect-modification evidence	Not used as independent primary main-effect estimate.
Zheng et al. (2017)	Hepatic	HCC; high MC-LR and high aflatoxin	HCC	428	Serum MC-LR; aflatoxin co-exposure	Biomonitoring-based serum MC assessment	Interaction estimate	OR	6.80 (95% CI 2.70–17.50)	Interaction: aflatoxin and MC-LR	Effect-modification evidence	Reported as interaction/co-exposure evidence; not used as primary estimate.
Feng et al. (2023)	Metabolic	Dyslipidemia; age <53 years subgroup	Dyslipidemia	720	Serum MCs	ELISA	Subgroup estimate	OR	4.12 (95% CI 2.01–8.44)	Stratified analysis by age	Effect-modification evidence	Dependent on parent study.
Feng et al. (2023)	Metabolic	Dyslipidemia; BMI <24 subgroup	Dyslipidemia	720	Serum MCs	ELISA	Subgroup estimate	OR	3.01 (95% CI 1.16–7.83)	Stratified analysis by BMI	Effect-modification evidence	Dependent on parent study.
Feng et al. (2023)	Metabolic	Dyslipidemia; female subgroup	Dyslipidemia	219	Serum MCs	ELISA	Subgroup estimate	OR	1.78 (95% CI 0.60–5.27)	Stratified analysis by sex	Effect-modification evidence	Non-significant subgroup estimate.

Study	Outcome group	Specific outcome	Outcome definition / ICD code, if reported	N	Exposure matrix	Analytical method	Estimate type	Effect metric	Adjusted estimate	Key covariates	Synthesis role	Notes
Feng et al. (2023)	Metabolic	Dyslipidemia; male subgroup	Dyslipidemia	501	Serum MCs	ELISA	Subgroup estimate	OR	3.30 (95% CI 1.68–6.48)	Stratified analysis by sex	Effect-modification evidence	Dependent on parent study.
Feng et al. (2023)	Metabolic	Dyslipidemia; high income subgroup	Dyslipidemia	720	Serum MCs	ELISA	Subgroup estimate	OR	4.38 (95% CI 1.70–11.26)	Stratified analysis by income	Effect-modification evidence	Dependent on parent study.
Feng et al. (2023)	Metabolic	Dyslipidemia; current/former smoking subgroup	Dyslipidemia	342	Serum MCs	ELISA	Subgroup estimate	OR	4.06 (95% CI 1.67–9.92)	Stratified analysis by smoking status	Effect-modification evidence	Dependent on parent study.
Feng et al. (2023)	Metabolic	Dyslipidemia; high MCs and high Zn	Dyslipidemia	720	Serum MCs; Zn co-exposure	ELISA; metal analysis	Interaction estimate	OR	2.18 (95% CI 1.26–3.77)	Interaction analysis: MCs and Zn	Effect-modification evidence	Do not count as independent main-effect estimate.
Feng et al. (2024b)	Metabolic	Metabolic syndrome; WBC level, ln-transformed 1-unit increase	Metabolic syndrome	591	MC-LR exposure	Biomonitoring-based exposure assessment	Mediation estimate	OR	3.70 (95% CI 1.74–7.86)	Mediation analysis using WBC	Mediation evidence	Mediation evidence from the same parent metabolic-syndrome study; not used in the primary meta-analysis.
Lin et al. (2023)	Metabolic	Gestational diabetes; age >30 subgroup	O24.4; gestational diabetes	Not reported	Serum MC-LR	Biomonitoring-based serum MC assessment	Subgroup estimate	OR	2.57 (95% CI 1.01–6.49)	Stratified analysis	Effect-modification evidence	Dependent on parent study.

Study	Outcome group	Specific outcome	Outcome definition / ICD code, if reported	N	Exposure matrix	Analytical method	Estimate type	Effect metric	Adjusted estimate	Key covariates	Synthesis role	Notes
Lin et al. (2023)	Metabolic	Gestational diabetes; income <10,000 RMB subgroup	O24.4; gestational diabetes	Not reported	Serum MC-LR	Biomonitoring-based serum MC assessment	Subgroup estimate	OR	2.63 (95% CI 1.13–6.11)	Stratified analysis	Effect-modification evidence	Dependent on parent study.
Lin et al. (2023)	Metabolic	Gestational diabetes; junior college and above subgroup	O24.4; gestational diabetes	Not reported	Serum MC-LR	Biomonitoring-based serum MC assessment	Subgroup estimate	OR	2.40 (95% CI 1.27–4.54)	Stratified analysis	Effect-modification evidence	Dependent on parent study.
Lin et al. (2023)	Metabolic	Gestational diabetes; parity ≥1 subgroup	O24.4; gestational diabetes	Not reported	Serum MC-LR	Biomonitoring-based serum MC assessment	Subgroup estimate	OR	2.97 (95% CI 1.03–8.59)	Stratified analysis	Effect-modification evidence	Dependent on parent study.
Lin et al. (2023)	Metabolic	Gestational diabetes; no passive smoking subgroup	O24.4; gestational diabetes	Not reported	Serum MC-LR	Biomonitoring-based serum MC assessment	Subgroup estimate	OR	2.69 (95% CI 1.32–5.46)	Stratified analysis	Effect-modification evidence	Dependent on parent study.
Gao et al. (2023)	Renal	CKD; high MCs and high As	CKD	270	Serum/plasma MCs; arsenic co-exposure	ELISA; ICP-MS	Joint-exposure estimate	OR	4.14 (95% CI 1.97–8.73)	Hypertension; reference: low MCs and low As	Co-exposure/effect-modification evidence	Do not treat as independent primary MC main effect.
Gao et al. (2023)	Renal	CKD; high MCs and low As	CKD	270	Serum/plasma MCs; arsenic co-exposure	ELISA; ICP-MS	Joint-exposure estimate	OR	1.40 (95% CI 0.64–3.09)	Hypertension; reference: low MCs and low As	Co-exposure/effect-modification evidence	Non-significant joint-exposure contrast.

Study	Outcome group	Specific outcome	Outcome definition / ICD code, if reported	N	Exposure matrix	Analytical method	Estimate type	Effect metric	Adjusted estimate	Key covariates	Synthesis role	Notes
Feng et al. (2022)	Renal	CKD; high MCs and high Cd	CKD	270	Serum/plasma MCs; cadmium co-exposure	ELISA; metal analysis	Joint-exposure estimate	OR	6.56 (95% CI 2.46–17.51)	Hypertension; reference: low MCs and low Cd	Co-exposure/effect-modification evidence	Reported prominently but excluded from primary main-effect pooling.

Part D. Sensitivity, review-derived, surveillance, and contextual estimates excluded from primary model

Study	Outcome group	Specific outcome	Outcome definition / ICD code, if reported	N	Exposure matrix	Analytical method	Estimate type	Effect metric	Adjusted estimate	Key covariates	Synthesis role	Notes
Gao et al. (2023)	Renal	CKD; high MCs using eGFR <60 definition	CKD	270	Serum/plasma MCs	ELISA; ICP-MS	Sensitivity estimate	OR	6.00 (95% CI 1.85–19.48)	Alternative outcome definition	Sensitivity evidence	Excluded from primary model; used for robustness interpretation.
Xu et al. (2020)	Renal	Renal injury; abnormal BUN, high vs low MC daily intake	Renal injury biomarker	5,493; review-derived parent population	Review-derived daily intake	Systematic review	Review-derived estimate	OR	1.80 (95% CI 1.34–2.42)	Not applicable	Excluded/contextual evidence	Review-derived estimate; retained only as contextual renal evidence and not pooled with primary human epidemiological studies.

Study	Outcome group	Specific outcome	Outcome definition / ICD code, if reported	N	Exposure matrix	Analytical method	Estimate type	Effect metric	Adjusted estimate	Key covariates	Synthesis role	Notes
Xu et al. (2020)	Renal	Renal injury; abnormal serum creatinine, high vs low MC daily intake	Renal injury biomarker	5,493	Review-derived daily intake	Systematic review	Review-derived estimate	OR	4.58 (95% CI 2.92–7.21)	Not applicable	Excluded/contextual evidence	Review-derived; not pooled with primary human epidemiological studies.
Xu et al. (2020)	Renal	Renal injury; abnormal eGFR, high vs low MC daily intake	Renal injury biomarker	5,493	Review-derived daily intake	Systematic review	Review-derived estimate	OR	4.41 (95% CI 2.55–7.63)	Not applicable	Excluded/contextual evidence	Review-derived; not pooled with primary human epidemiological studies.
Bloch et al. (2024)	Contextual/systemic	Cyanobacteria harmful algal bloom exposure reports; systemic acute illness	T65.8; systemic toxic effects/acute illness	4,260 exposure reports	Recreational/drinking-water exposure reports	Poison-center surveillance/clinical report	Surveillance/contextual estimate	IRR	1.40 (95% CI 1.12–1.75)	Not applicable	Contextual public-health evidence	Acute exposure surveillance; excluded from chronic hepatic/renal/metabolic primary model.

Note: MC, microcystin; OR, odds ratio; RR, risk ratio; HR, hazard ratio; IRR, incidence rate ratio; CI, confidence interval; LC–MS/MS, liquid chromatography–tandem mass spectrometry; ELISA, enzyme-linked immunosorbent assay; RoB, risk of bias; HBV, hepatitis B virus; HCC, hepatocellular carcinoma; CKD, chronic kidney disease. The primary synthesis prioritized the most fully adjusted main population-level estimate for each study–outcome group. Only estimates classified as primary main estimates were used in the primary outcome-specific quantitative synthesis. Dose-response, subgroup, interaction, co-exposure, mediation, sensitivity, review-derived, surveillance, and contextual estimates were retained for secondary interpretation only and were not treated as independent primary observations. These estimates are reported in this table according to synthesis role to support transparency in effect-size selection and interpretation. Estimates duplicated across primary and dose-response sections, such as selected highest-category estimates, were counted only once in the primary quantitative synthesis. ICD codes are shown only when reported or clearly used in the source study. For biomarker-defined, lipid-profile-defined, or study-criteria-defined outcomes, the original outcome definition is reported without assigning an additional ICD code.

Table S6. Risk-of-bias assessment for primary epidemiological estimates.

Risk-of-bias domains were assessed for the seven independent human epidemiological estimates included in the primary outcome-specific quantitative synthesis. Judgments were assigned at the study–outcome estimate level across participant selection, exposure assessment, outcome assessment, confounding control, temporality, reporting, and overall risk of bias. L, low risk; M, moderate or probably high concern; H, high risk; NA, not applicable.

**Study ID**	**Evidence domain**	**Outcome group**	**Selection**	**Exposure**	**Outcome**	**Confounding**	**Time-related**	**Reporting**	**Overall**
Li et al. (2011)	P1 Epidemiology	Hepatic	M	M	M	M	M	L	Moderate
Zheng et al. (2017)	P1 Epidemiology	Hepatic	M	L	L	M	M	L	Low–moderate
Gao et al. (2023)	P1 Epidemiology	Renal	L	M	M	M	M	L	Low–moderate
Feng et al. (2023)	P1 Epidemiology	Metabolic	L	M	M	M	M	L	Low–moderate
Feng et al. (2024b)	P1 Epidemiology	Metabolic	M	M	M	M	M	L	Moderate
Lin et al. (2023)	P1 Epidemiology	Metabolic	L	M	L	M	M	L	Moderate
Zeng et al. (2017)	P1 Epidemiology	Metabolic	M	M	M	M	M	L	Moderate

Note. Risk-of-bias judgments were assigned to the primary study–outcome estimates used in the one-study–one-main-estimate synthesis. Exposure-assessment concerns reflect differences in exposure matrix, analytical specificity, exposure timing, and potential exposure misclassification. Confounding concerns reflect incomplete or heterogeneous control of outcome-specific covariates, including hepatitis virus infection, aflatoxin exposure, alcohol use, body mass index, hypertension, diabetes, socioeconomic factors, and metal or contaminant co-exposures. Time-related concerns reflect the predominance of cross-sectional, case-control, or nested case-control designs and limited longitudinal evidence. Mechanistic, environmental, governance, economic, and vulnerability-related studies were treated as contextual evidence and were not included in the primary quantitative risk-of-bias table.

Table S7. Selected international guideline and advisory values for microcystins in drinking and recreational waters Selected jurisdiction-specific values for microcystins are summarized by analyte, water matrix, and regulatory or advisory basis. Values are standardized to µg/L. Interpretation should consider differences in analyte definition, exposure duration, monitoring triggers, analytical capacity, and regulatory enforceability.

**Jurisdiction**	**Guideline / advisory value (µg/L)**	**Analyte**	**Matrix**	**Regulatory notes / basis**
WHO	1.0	MC-LR / microcystins, expressed using MC-LR toxicological basis	Drinking water	Provisional lifetime drinking-water guideline value; WHO also provides short-term drinking-water and recreational-water values.
U.S. EPA	0.3 for infants and preschool-aged children; 1.6 for school-aged children and adults	Total microcystins	Drinking water	10-day Health Advisories; non-regulatory advisory values.
U.S. EPA	8.0	Total microcystins	Recreational water	Recommended recreational ambient water-quality criterion or swimming advisory value.
European Union	1.0	Microcystin-LR	Drinking water	Directive (EU) 2020/2184 parametric value; measured when cyanobacterial blooms are likely or present in source water.
Republic of Korea	1.0	MC-LR	Drinking water	National drinking-water management value aligned with the WHO provisional value.
Australia / New Zealand	≈1.3	Total microcystins or MC-LR toxicity-equivalent basis, depending on guidance application	Drinking water	National guidance value; interpretation depends on analytical and toxicity-equivalence conventions used.
Canada	1.5	Total microcystins	Drinking water	Health Canada maximum acceptable concentration for total microcystins in drinking water.

Note. Values differ across jurisdictions because they are based on different analyte definitions, exposure durations, populations, regulatory triggers, and advisory frameworks. The table is provided for contextual comparison of monitoring and risk-management thresholds only. These values were not used in the primary meta-analysis, certainty assessment, or causal inference.

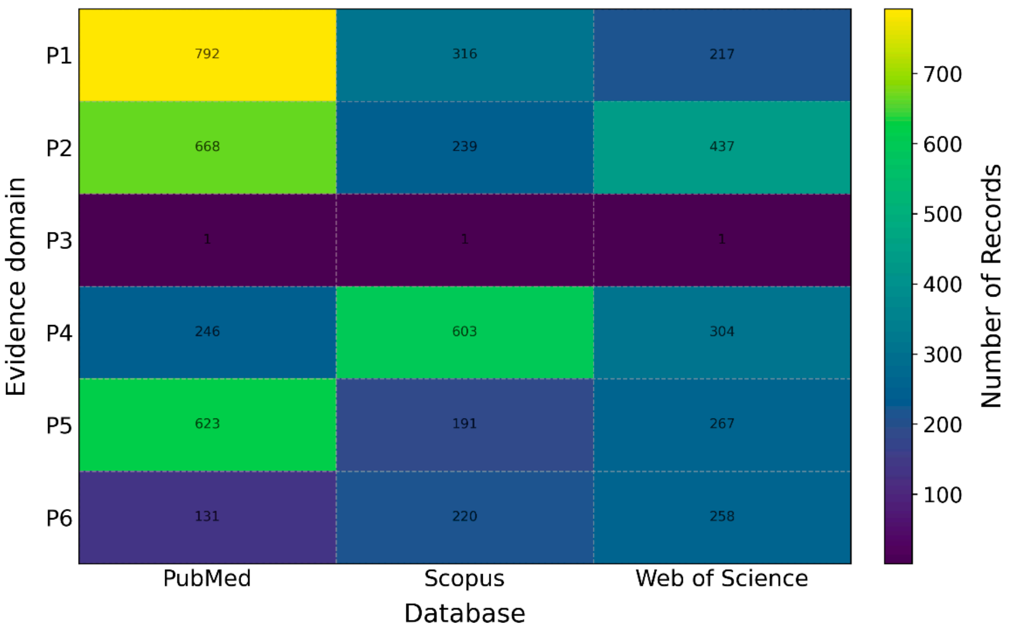

Figure S1. Evidence-density heatmap by contextual evidence domain and bibliographic database.

Heatmap showing evidence-domain mapped record counts in PubMed, Scopus, and Web of Science across P1–P6. The cells reflect the records assigned to each database–domain combination during evidence mapping. They are descriptive mapping counts and are not equivalent to the mutually exclusive raw database totals used in the PRISMA identification stage. Records could be identified through broad and targeted searches and subsequently assigned to an evidence domain; therefore, the heatmap should be interpreted as the density of the mapped evidence base rather than as a reconstruction of the 9,021 pre-deduplication records.

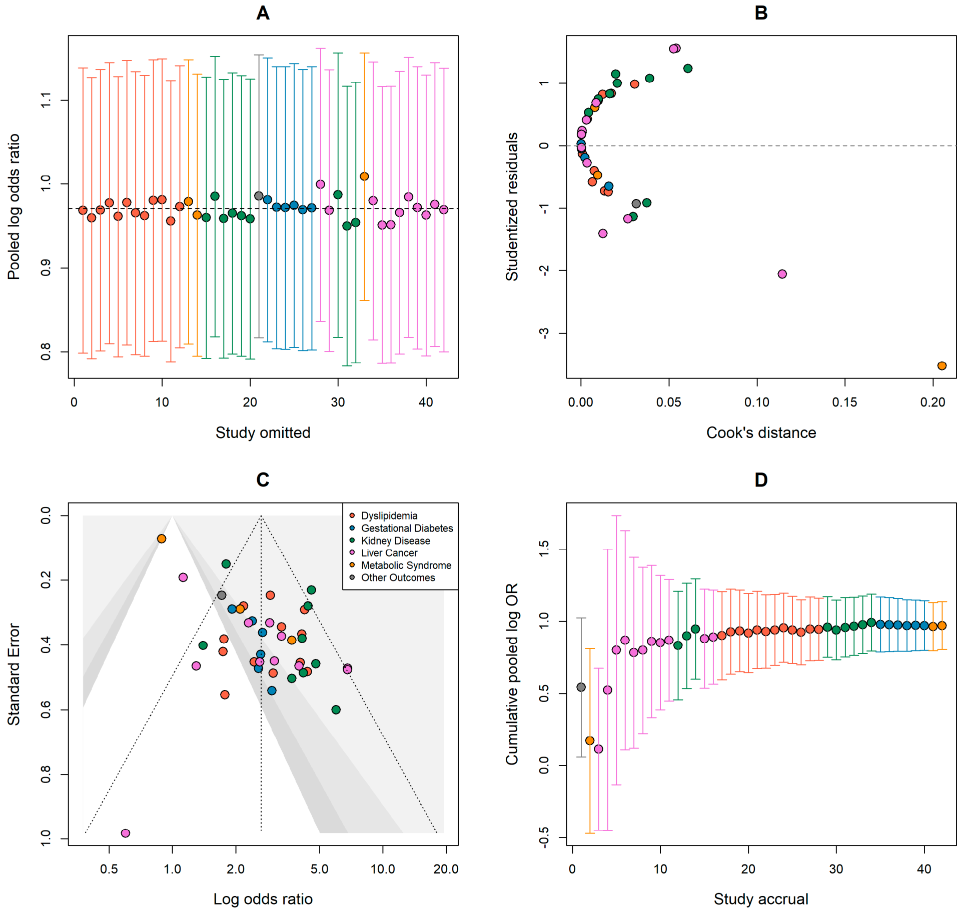

Figure S2. Exploratory only— Sensitivity and influence diagnostics for the expanded extracted-estimate comparison.

Diagnostic plots for the expanded extracted-estimate comparison: (A) leave-one-out analysis of pooled log odds ratios, (B) influence diagnostics based on Cook's distance and studentized residuals, (C) contour-enhanced funnel plot, and (D) cumulative meta-analysis by study accrual. These diagnostics were used to examine robustness, influence, small-study effects, and heterogeneity within the broader extracted-estimate set. Because this expanded dataset includes statistically dependent estimates derived from some parent studies, these diagnostics were interpreted only as secondary exploratory evidence and were not used as the primary inference.

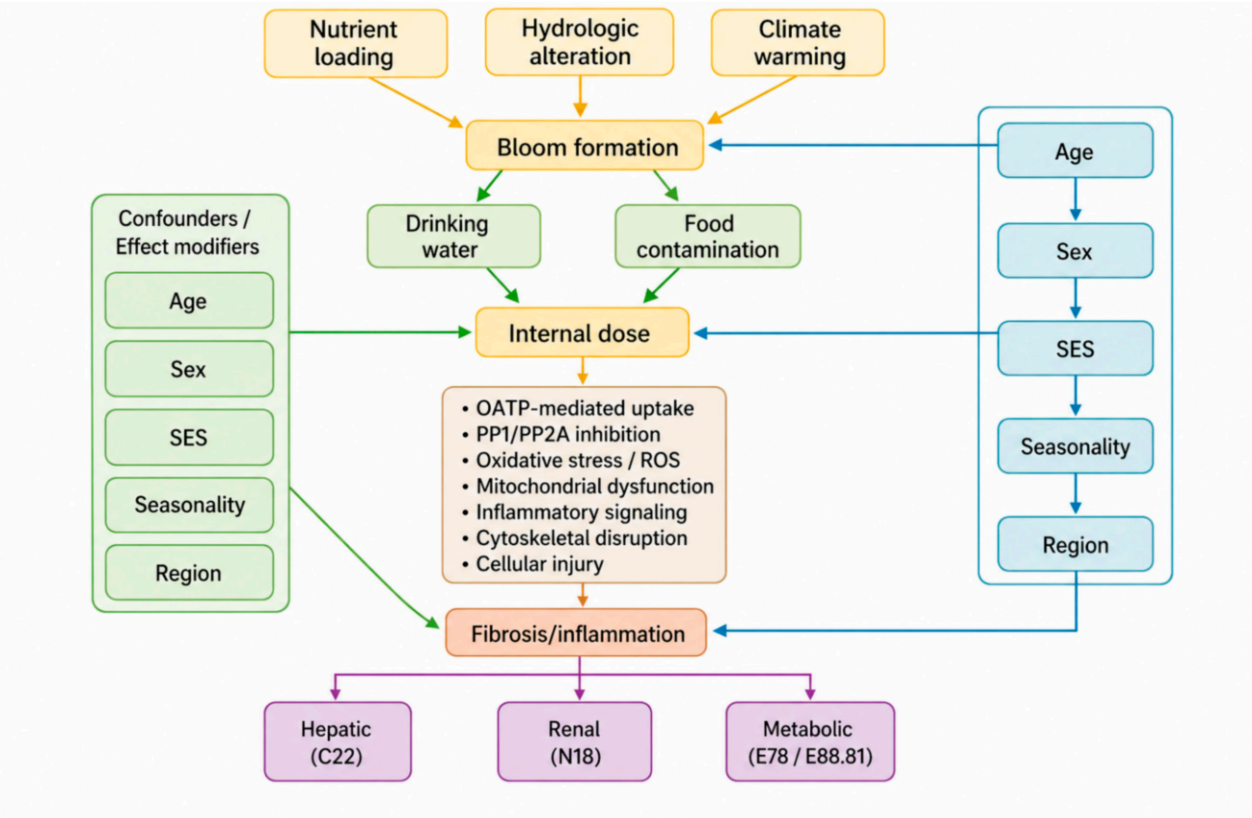

Figure S3. Integrated directed acyclic graph for chronic microcystin exposure and hepatic, renal, and metabolic outcomes.

The directed acyclic graph links upstream environmental drivers, including nutrient loading, hydrologic alteration, and climate warming, to cyanobacterial bloom formation, exposure pathways, internal dose, mechanistic mediators, and the outcome groups evaluated in this review. Drinking-water and food-web exposure pathways are shown as representative routes leading to internal dose. Mechanistic mediators include OATP-mediated uptake, PP1/PP2A inhibition, oxidative stress, mitochondrial dysfunction, inflammatory signaling, and related cellular injury pathways. The outcome nodes correspond to hepatic (C22), renal (N18), and metabolic (E78/E88.81) endpoints. Host and contextual factors, including age, sex, socioeconomic status, seasonality, and region, are represented as potential confounders or effect modifiers. Governance and infrastructure nodes indicate surveillance-relevant intervention points, including monitoring capacity, treatment capacity, analytical infrastructure, and regulatory enforcement. DAG was used for conceptual organization and confounder identification and was not treated as independent causal evidence.

Source note. Study identifiers and guideline sources are reported directly in Supplementary Tables S5–S7. No separate supplementary reference list is provided; bibliographic references cited in the article are included in the main manuscript reference list.
