# Supplementary material for: Human Health Effects of Chronic Microcystin Exposure: A Systematic Review and Meta-Analysis of Hepatic, Renal, and Metabolic Outcomes
Source: Toxins (Basel). 2026 Aug 30;18(9):373. doi: 10.3390/toxins18090373 (PMC13611463; doi:10.3390/toxins18090373)
Supplement: Supplementary file 1 [file toxins-18-00373-s001.zip › toxins-4516077_Supplementary_Materials_2.pdf]

PRISMA 2020 Checklist

Human Health Effects of Chronic Microcystin Exposure: A Systematic Review and Meta-Analysis of Hepatic , Renal, and Metabolic Outcomes

Locations refer to the revised Toxins manuscript and revised Supplementary Materials; section references are used because pagination may change during production.

Section and Topic	Item #	Checklist Item	Location Where Reported
TITLE	**1**	Identify the report as a systematic review.	Title
ABSTRACT	**2**	Report an abstract addressing the PRISMA 2020 for Abstracts items.	Abstract; see abstract checklist below
INTRODUCTION	**3**	Describe the rationale for the review in the context of existing knowledge.	Introduction
INTRODUCTION	**4**	Provide an explicit statement of the review objectives or questions.	Introduction
METHODS	**5**	Specify inclusion and exclusion criteria and how studies were grouped for syntheses.	Sections 5.1 and 2.3
METHODS	**6**	Specify all information sources searched or consulted and the date each source was last searched.	Section 5.2; Supplementary Table S2
METHODS	**7**	Present the full search strategies for all databases, including filters and limits.	Supplementary Table S2
METHODS	**8**	Specify the methods used to decide whether a study met the inclusion criteria, including reviewer independence.	Section 5.3
METHODS	**9**	Specify the methods used to collect data from reports, including reviewer independence.	Section 5.3
METHODS	**10a**	List and define all outcomes for which data were sought.	Sections 5.1 and 2.3 and 6–7; Supplementary Table S4
METHODS	**10b**	List and define all other variables for which data were sought and assumptions made for missing information.	Section 5.3; Supplementary Table S4
METHODS	**11**	Specify the methods used to assess risk of bias in included studies.	Section 5.4; Supplementary Table S6
METHODS	**12**	Specify the effect measure(s) used for each outcome.	Section 5.5
METHODS	**13a**	Describe the process used to decide which studies were eligible for each synthesis.	Sections 5.3 and 2.5
METHODS	**13b**	Describe methods used to prepare data for presentation or synthesis.	Section 5.5
METHODS	**13c**	Describe methods used to tabulate or visually display results.	Section 5.5; Tables 1–3; Figures 1–4
METHODS	**13d**	Describe methods used to synthesize results and justify model choices, heterogeneity methods, and software.	Section 5.5
METHODS	**13e**	Describe methods used to explore possible causes of heterogeneity.	Section 5.5
METHODS	**13f**	Describe sensitivity analyses conducted to assess robustness.	Section 5.5
METHODS	**14**	Describe methods used to assess risk of bias due to missing results.	Section 5.5; formal testing restricted to models with at least 10 estimates
METHODS	**15**	Describe methods used to assess certainty or confidence in the body of evidence.	Section 5.4
RESULTS	**16a**	Describe the results of the search and selection process, ideally using a flow diagram.	Section 2.1 and Figure 1
RESULTS	**16b**	Cite studies that appeared to meet inclusion criteria but were excluded and explain why.	Aggregate full-text exclusion reasons are reported in Figure 1. A study-level exclusion log was unavailable; this limitation is stated in Section 3.5 and the Supplementary Materials.
RESULTS	**17**	Cite each included study and present its characteristics.	Section 2.2; Table 1; Supplementary Table S5
RESULTS	**18**	Present risk-of-bias assessments for each included study.	Table 1; Supplementary Table S6
RESULTS	**19**	For all outcomes, present results for each study, including effect estimates and precision.	Table 1; Supplementary Table S5
RESULTS	**20a**	For each synthesis, summarise the characteristics and risk of bias of contributing studies.	Sections 2.2–3.4; Tables 1–2
RESULTS	**20b**	Present results of all statistical syntheses, including summary estimates, precision, and heterogeneity.	Section 2.4; Figure 2; Table 2
RESULTS	**20c**	Present results of investigations of possible causes of heterogeneity.	Section 2.4; narrative interpretation because subgroup sizes were limited
RESULTS	**20d**	Present results of sensitivity analyses.	Section 2.4; Supplementary Figure S2
RESULTS	**21**	Present assessments of risk of bias due to missing results.	Section 2.4 and Supplementary Figure S2; formal small-study-effect testing was limited by model size
RESULTS	**22**	Present assessments of certainty or confidence for each outcome.	Table 2; Discussion
DISCUSSION	**23a**	Provide a general interpretation of results in the context of other evidence.	Sections 3.1–4.4
DISCUSSION	**23b**	Discuss limitations of the evidence included in the review.	Sections 3.1–4.5
DISCUSSION	**23c**	Discuss limitations of the review processes used.	Section 3.5
DISCUSSION	**23d**	Discuss implications for practice, policy, and future research.	Sections 3.4–4.5, Sections 3.4–3.5 and Conclusions
OTHER INFORMATION	**24a**	Provide registration information or state that the review was not registered.	Section 5.1: no prospective public protocol was registered
OTHER INFORMATION	**24b**	Indicate where the review protocol can be accessed or state that a protocol was not prepared.	Section 5.1: no prospective protocol was prepared; reconstructed methods are documented in the Supplementary Materials

OTHER INFORMATION	**24c**	Describe and explain any amendments to registration or protocol information.	Not applicable because no prospective registration or protocol was available
OTHER INFORMATION	**25**	Describe sources of financial or non-financial support and the role of funders.	Funding statement
OTHER INFORMATION	**26**	Declare competing interests.	Conflicts of Interest statement
OTHER INFORMATION	**27**	Report which materials are publicly available and where they can be found.	Data Availability Statement; revised Supplementary Materials

PRISMA 2020 for Abstracts Checklist

Item #	Abstract Checklist Item	Location Where Reported
1	Identify the report as a systematic review.	Title
2	Provide an explicit statement of the main objective(s) or question(s).	Abstract
3	Specify the inclusion and exclusion criteria for the review.	Abstract; detailed criteria in Sections 5.1 and 2.3
4	Specify the information sources and date of the last search.	Abstract: PubMed, Scopus, Web of Science; update search April 2026
5	Specify the methods used to assess risk of bias in included studies.	Abstract
6	Specify the methods used to present and synthesize results.	Abstract: outcome-specific synthesis and one-study–one-main-estimate rule
7	Give the total number of included studies and participants and summarise relevant study characteristics.	Abstract: 192 studies, 34 human studies, 7 estimates; unduplicated participant total not reported because it could not be reliably derived
8	Present results for the main outcomes, preferably with number of studies and summary estimates with precision.	Abstract
9	Provide a brief summary of limitations of the evidence.	Abstract: renal evidence limited; metabolic evidence heterogeneous
10	Provide a general interpretation of results and important implications.	Abstract
11	Specify the primary source of funding.	Not stated in Abstract; Funding statement
12	Provide the register name and registration number, or state that the review was not registered.	Abstract: review was not prospectively registered

Note: Item 16b remains only partially reported because an itemised list of near-miss full-text exclusions was not available in the retained review files. No exclusions were fabricated for this checklist.
